# The Molecular Mechanism of Action of the CR6261-Azichromycin Combination Found through Computational Analysis

**DOI:** 10.1371/journal.pone.0037790

**Published:** 2012-05-31

**Authors:** Wei Cui, Kui Wang, Jishou Ruan, Zhi Qi, Yi Feng, Yiming Shao, Jack A. Tuszynski

**Affiliations:** 1 College of Mathematical Sciences, LPMC, Nankai University, Tianjin, People's Republic of China; 2 National Center for AIDS/STD Control and Prevention, Chinese Center for Disease Control and Prevention, Beijing, People's Republic of China; 3 Division of Experimental Oncology, Cross Cancer Institute, Edmonton, Alberta, Canada; 4 State Key Laboratory for Medicinal Chemical Biology, Nankai University, Tianjin, People's Republic of China; Shanghai Medical College, Fudan University, China

## Abstract

**Background:**

CR6261 was found in 2008 and F10 was found in 2009. In 2010 Friesen et al experimentally showed that Oseltamivir/Zanamivir may improve the therapeutic efficacy of CR6261. As a result, the use of CR6261 combined with a drug to provide an antibody-based therapy against all influenza A viruses was proposed. Although CR8020 may neutralize group 2 influenza viruses and FI6 may neutralize both group 1 and group 2 influenza viruses as determined in 2011, the insight of Friesen et al is still interesting. Here, we address the following questions: how to uncover the molecular mechanism of a drug, which improves the therapeutic efficacy of mAbs and how to find drugs that enable CR6261 (CR8020, F10) to become a universal mAb.

**Methods and Findings:**

Using the 3D structures of 3 gbn, 3 gbm, 3 ztn, 3 ztj, 3 fku and 3 sdy, we separate the 3D structures of CR6261, F10, CR8020 and FI6, and the 3D structures of trimer HAs of H3N2 and H5N1. Based on the experimental result of Friesen et al, we have found many clues, which reveal the molecular mechanism of action for a drug and an HA-mAb complex.

**Conclusions:**

Oseltamivir/Zanamivir may congruously improve the therapeutic efficacies of CR6261, F10, CR8020 and FI6 by providing an additional affinity to compensate for the loss of affinity between HA and mAb resulting from mutations. However, Oseltamivir or Zanamivir are not expected to generally widen the spectrum of these mAbs. In order to enhance CR6261, CR8020, or for F10 to become universal, we may select Azichromycin, Oseltamivir, or the combination of Azichromycin and Oseltamivir, respectively.

## Introduction

### General Background

Since the discovery of the human monoclonal antibody CR6261 published by Throsby et al ([1], PLoS ONE 2008), the isolation of an impressively wide spectrum of antibodies and therefore a family of monoclonal antibodies (mAbs) was made possible, e.g. F10 ([2], Sui et al, Nat Struct Mol Biol, 2009), CR8020 ([3] Ekiert et al, Science 2011), FI6 ([4], Corti et al, Science 2011). It was determined that (a) CR6261 and F10 may neutralize all group 1 influenza viruses, (b) CR8020 may neutralize all group 2 influenza viruses, and (c) FI6 is the unique mAb to neutralize both group 1 and group 2 influenza A viruses. The discovery of mAbs is the prime mover in the development of new vaccines and antibody-based therapies. For example, an exploration of improved universal vaccines for all influenza A viruses based on CR6261-like antibodies was proposed in the papers by Wei et al ([5], Science 2010) and Nabel et al ([6], Nature 2010). Also, Friesen et al evaluated the prophylactic and therapeutic efficacy of the CR6261 antibody against a lethal challenge due to the highly pathogenic avian H5N1 virus in ferrets ([7], PLoS ONE 2010). They further provided the insight that the use of CR6261 in combination with an effective drug (i.e., Oseltamivir or Zanamivir) could become an antibody-based therapy against all influenza A viruses. These studies have defined a new paradigm in the research on vaccines and provided a useful starting point for the design of new vaccines.

Although a universal mAb FI6 has already been found, the insight for the use of a drug in a complex with CR6261 to neutralize all influenza A viruses is still worth pursuing, because it can provide a general method to enhance a wide spectrum of mAb and enable them to become a universal antibody. This can also show the way to enhance a universal mAb and avoid drug resistance. Therefore, this approach may lead to multiple choices for antibody-based therapies. The use of mAb in a combination with a drug will be easier and cheaper relative to the cocktail method that is based on two types of mAbs. Therefore, one of the objectives of this study is to provide a new insight regarding the utilization of mAbs. With an increasing number of mAbs becoming available, selectivity of mAbs in combination with drugs offers an opportunity to construct better mAb-drug combinations.

In this paper, we first determine the molecular mechanism by which Oseltamivir and Zanamivir improve the therapeutic efficacy of an mAb. Then, we look for the drugs which enhance CR6261, F10 or CR8020 to become a universal mAb, respectively. To perform the latter task, we must first deal with the hard problem of demonstrating the relationship between mAbs and the trimer HAs while being fully aware of the fact that mAbs cannot neutralize influenza viruses. For example, since we know that CR6261 cannot neutralize all group 2 influenza viruses, we should show that CR6261 and group 2 HAs may be combined first.

The fact that Oseltamivir or Zanamivir in complex with CR6261 improves the therapeutic efficacy of CR6261 to treat group 1 influenza viruses is a crucial piece of evidence in support of the assumption that Oseltamivir must directly act on either 3 gbn or 3 gbm. In fact, we cannot use the cocktail idea to explain this enhancement of the therapeutic efficacy of CR6261 by adding Oseltamivir. This is because Oseltamivir is ineffective against H5N1 when it binds to the NA protein of H5N1 and Oseltamivir does not bind to the trimer HA alone. Therefore, the complexed protein (CR6261 with the trimer H5 HA) must be the molecular target for the action of Oseltamivir. For the same reason, we know that the CR6261-trimer HA (for all group 1 HAs) must be the target protein for both Oseltamivir and Zanamivir. In order to uncover the mechanism by which a drug may improve the therapeutic efficacy of CR6261, we should look for a clue from the complex CR6261-trimer HA for all group 1 HAs. The result that Oseltamivir may improve the therapeutic efficacy of CR6261 in treatment of H5N1 should also lead to additional clues. For example, the docking poses between the drug and its benchmark pockets are known to be very diverse.

Other clues and additional information will be gradually extracted. For example, based on CR8020, we find that the footprint of mAb containing an epitope is not a necessary condition for mAb to neutralize influenza viruses. This clue leads us to focus on the issue of affinity. However, it is clear that an antibody is ineffective if its footprint is located on the head of a trimer HA. To look for underlying clues, one should use both computational tools and experimental methods. Since this is not an experimental project, we will use computational modeling to the greatest extent possible.

Based on all clues deduced from the well-known complexes of mAbs and HAs, we determine a general molecular mechanism to explain why a drug may improve the therapeutic efficacy of CR6261, CR8020, F10 and FI6. In order to look for a drug used in combination with CR6261, F10 or CR8020 to enable it to become a universal mAb, or enhance the effect of FI6 to prevent drug resistance, we need additional insights. They can be used to show that CR6261 and F10 bind to group 2 HAs and to predict the location of the footprints of CR6261 (F10) on H3 HA.

### Tools and Materials Used

First, we use computational methods, namely protein-protein docking algorithms to show that CR6261 also bind to group 2 HAs. Clearly, it would be ideal if all spatial structures of group 2 HAs were well known. However, only 3D structures of H3 HA and H7 HA are available in the Protein Data Bank (PDB). This small number of structures is obviously insufficient. Fortunately, the conservation of footprints of antibodies on each of the group 2 HAs ensures that we may be able to infer a general conclusion based on some representative cases.

The available crystal structures of HAs in a complex with an antibody are 3 gbn (the H1 HA in complex with CR6261, [8]), 3 gbm (the H5 HA in complex with CR6261, [8]), 3 sdy (the H3 HA in complex with CR8020, [3]), 3 fku (the H5 HA in complex with F10, [2]), 3 ztj (the H3 HA in complex with FI6, [4]) and 3 ztn (the H1 HA in complex with FI6, [4]). These six crystal structures form the dataset representing prior knowledge giving us insights, which will be used to uncover the molecular mechanism of action. For example, based on 3 gbn and 3 gbm, we find that the footprints of CR6261 on H1 and H5 are the same. Based on 3 ztn and 3 ztj, we find that the footprints of CR6261 on H1 and H3 are not the same. Based on 3 fuk and 3 gbm, or 3 sdy and 3 ztj, we find that the footprints of CR6261, F10 and FI6 or CR8020 and F10 on the same HAs are different. In particular, the footprint of CR8020 challenges the notion that a footprint must contain an epitope including a fusion peptide and an αA helix.

The conservation of a footprint helps us find the common conserved area on the surface of a stem of group 1 HAs or group 2 HAs. This conserved area is larger than the union of the footprints from a wide spectrum of mAbs on group 1 HAs or group 2 HAs. For this purpose, we need to use multiple sequence alignment algorithms and the benchmark dataset of all available HA sequences. Since public algorithms [9]–[17] do not work well for large-size datasets, we have decided to use a new multiple sequence alignment algorithm MCABMSA (introduced in Section 4 of Information S1). Using MCABMSA, we have generated a dendrogram of H1–H16 and the consensus sequences of H1–H16. According to the five-group method used in ref. [2], we carry out a conservation analysis of residues in a site-by-site fashion. As a result, we have been able to construct the conserved area on the surface of group 1 HAs and group 2 HAs, respectively.

In order to look for the binding pocket of Oseltamivir/Zanamivir on the target protein 3 gbn, we use AutoDock [18]–[22]. It is a good tool for finding the binding pocket of a drug on a given target protein. We have no reason to doubt the quality of AutoDock. However, having performed large-scale validation exercises, we noticed that AutoDock has a tendency to be trapped in local energy minima. As a consequence, it appears that AutoDock often sends ligands to the same area where all input drugs arrive at their minimum free energy locations. Our large-scale validation is shown in Section 1 of Information S1. This specific property encourages us to utilize this software to search for a binding pocket using a panel of drugs. For different cases studied, we have assembled different panels of drugs. In general, the panel of drugs used should have a balanced distribution with an appropriate rate between positive and negative controls. In this paper, the panel of nine drugs used consists of Amantadine, Azithromycin, Aspirin, HEM, Heroin, Isosorbide, Oseltamivir, Zanamivir, and Vancomycin. Details regarding the choice of the panel of drugs are given in Section 2 of Information S1. How to explore the binding pocket on a given target protein based on a panel of drugs is stated in Section 1 of Information S1.

Although CR6261 was validated experimentally to neutralize group 1 influenza A viruses, its molecular mechanism of action has not been uncovered yet. To determine the mechanism, we analyze all 3D structures of the HA-CR6261 complex for all group 1 HAs. This is because we can use it to obtain a critical affinity for CR6261 neutralizing group 1 influenza viruses. Nevertheless, we have only used 3 gbn with 3 gbm, which are partial crystal structures of HA-CR6261 complexes for H1 HA and H5 HA, respectively. As additional support, we have used all crystal structures 3 gbn, 3 gbm, 3 fuk, 3 sdy, 3 ztn and 3 ztj as a test panel to estimate the critical affinity for an mAb neutralizing the influenza viruses. Then, we have used the Ligand Explorer software and the molecular dynamic simulation (MD) software (e.g., GROMACS) to estimate the upper bound of the binding free energy for an antibody to unbind from an HA. As an introduction to MD simulations, we refer the reader to ref. [23].

As approximate models of the crystal structure of CR6261 or F10 in complex with H3 HA, we use the predicted 3D structures. For this purpose, we need to use the protein-protein docking software (RosettaDock). As an introduction to the topic of protein-protein docking, we recommend refs. [24]–[26].

Before we discuss the results obtained, we need to explain the benchmark dataset in more detail. We first downloaded all sequences of influenza A viruses from the Uniprot database updated on Dec.6, 2010. We then retrieved all HA sequences. All A-type sequences were classified into 16 types, denoted by H1, H2, etc., up to H16. To avoid confusion, we simply denoted C-type HA sequences and B-type sequences as HC and HB, respectively. The total number of HA sequences in the benchmark dataset is 36,051 (some sequences of a mixed or unidentified type have not been deleted from this dataset) and we have used these 36,051 sequences as the benchmark dataset in this paper. Section 3 of Information S1 shows how to obtain this dataset in detail.

Since we have used the unpublished multiple sequence alignment software MCABMSA, it is worth explaining the reason for it. Numerous software packages are readily available for use, for example, BLAT [9], AVID [10], MUSCLE [11], COFFEE [12], SAGA [13], MAVID [14], MSAID [15], Mauve [16] and MAFFT [17]. However, the first 8 algorithms are slow and do not work well for datasets with 5,000 sequences or more. Comparably, MAFFT is very fast, but it also does not work well for datasets with 10,000 sequences or more. In the benchmark dataset, the number of sequences of H1 or H3 is equal or larger than 10,000. Therefore, we have decided to use the novel multiple sequence alignment software MCABMSA, whose full name is “Multiple Compressed and Anchor-Based Multiple Sequence Alignment for the Sequences of Viruses”. It involves four parameters: (1) the length of an anchor point, (2) the number of wrong letters that are tolerated, (3) the maximum relaxed number, and (4) the coverage rate of anchor points. A brief description of the function of each parameter and the advantage of MCABMSA is provided in Section 4 of Information S1.

## Materials and Methods

### Retrieving Side Information

It will be very convenient to directly use the dendrogram of H1–H16 used in refs. [2]–[6], which was constructed based on 39 representative sequences. Moreover, if we use these 39 representatives to analyze the conservation of residues of HAs in the five-group fashion [2], this will save us a large amount of space. We need to look for useful clues when reconstructing the dendrogram of H1–H16 and when reanalyzing the conservation of residues based on the sequences made available over the past 90 years. The numbers of all subtypes are listed in Table 1.

**Table 1:** The numbers of HA sequences for 18 cases in the benchmark dataset.

HC	HB	H1	H2	H3	H4	H5	H6	H7	H8	H9	H10	H11	H12	H13	H14	H15	H16
54	3002	9837	315	14235	467	4301	739	989	56	1354	201	145	63	69	7	10	24

### Retrieving Side Information by Reconstructing the Dendrogram

A convenient method to reconstruct the dendrogram of H1–H16 is to group all available sequences into a few clusters using MCABMSA under each given parameter *r*. We then transform each cluster as an 18-dimensional vector using the following steps:

1. Compute *r_k_  =  the number of the k-th type of HA sequences appearing in the cluster as a function of the number of the k-th type of HA sequences shown in* *Table 1*.
2. Transform the cluster into the vector *v  =  (r_1_,r_2_,r_3_,r_4_,…,r_18_)*.

Analyzing the components of each vector, we readily recognize which Hk sequences are grouped into the same cluster. For example, MCABMSA may output 7 clusters after being aligned under *r* = 0.001. Among the corresponding 7 vectors, only 5 vectors have the maximum components that are greater than 0.01 as listed in Table 2.

**Table 2:** The vectors induced from the five clusters.

	C	B	H1	H2	H3	H4	H5	H6	H7	H8	H9	H10	H11	H12	H13	H14	H15	H16
HA_1	**1.0000**	0.0000	0.0003	0.0000	**0.2952**	**0.8843**	**0.7901**	0.0000	0.0010	0.0000	0.0185	0.0000	0.0000	0.0000	0.0000	**0.8571**	0.0000	0.0000
HA_2	0.0000	**1.0000**	**0.7975**	**0.7922**	**0.1590**	0.0306	**0.1301**	**0.9318**	**0.9342**	**0.9800**	**0.8175**	**0.9353**	**0.9379**	**0.9661**	**0.9848**	**0.1429**	**0.9000**	**0.9583**
HA_3	0.0000	0.0000	**0.1619**	0.0519	**0.5203**	**0.0699**	0.0780	**0.0655**	**0.0648**	0.0000	0.0542	**0.0647**	0.0207	0.0339	0.0000	0.0000	**0.1000**	0.0417
HA_5	0.0000	0.0000	**0.0404**	**0.1558**	0.0000	0.0087	0.0019	0.0027	0.0000	0.0200	**0.0853**	0.0000	**0.0414**	0.0000	0.0152	0.0000	0.0000	0.0000
HA_6	0.0000	0.0000	0.0000	0.0000	**0.0256**	0.0066	0.0000	0.0000	0.0000	0.0000	**0.0245**	0.0000	0.0000	0.0000	0.0000	0.0000	0.0000	0.0000

In Table 2, HA_k denotes a vector corresponding to the cluster HA_k. For a given vector, each of the 18 components is the rate of the number of these sequences grouped into the cluster HA_k as a function of the total number of sequences as shown in Table 1. From Table 1, we find that most of H4, H5 and H14 sequences and a part of H3 sequences which share a common peptide with HC sequences. The second vector tells us that most of the H1, H2, H6, H7, H8, H9, H10, H12, H13, H15, H16 and HB sequences share a common peptide with HB sequences. The first and the second vectors jointly show that the HB and HC sequences have no common peptide. The third vector shows that a large number of H3 sequences have no peptide in common with both HC and HB sequences. These vectors not only give us an approximate relationship among these 18 classes of sequences, but also determine the common peptides, if we inversely track them from the cluster. For example, the common peptide shared by H1, H2, H6, H7, H8, H9, H10, H12, H13, H15, H16 and HB sequences is the palindrome peptide FGAIAGF.

To distance H1–H16 from HB and HC, we need to gradually enlarge the parameter *r*. As *r* > 0.2, we find that the HB and HC sequences are significantly separated from the H1–H16 sequences. Therefore, for consistency we select 0.4, 0.5, 0.6, 0.7 and 0.8. Then we have 6 groups of vectors formed. According to these 6 groups of vectors, we plot 6 figures. These 6 figures exactly show that progression of the dendrogram of H1–H16 as *r* increases equidistantly. At *r = *0.8 this dendrogram virtually coincides with that dendrogram used in refs. [2]–[6]. In other words, we have successfully reconstructed the dendrogram in a new way. For more details, the reader is referred to Section 5 of Information S1. The value of this exercise is to help identify which peptides play the role of important markers among the H1–H16 sequences. These peptides will be used as side information in order to find the molecular mechanism of action.

#### Retrieving side information by reanalyzing the conservation of residues

The main idea to reanalyze the conservation of the residues on the surface of HA using the five-group method based on all available sequences is stated as below:

1. Rebuild datasets of HA1 and HA2 based on the benchmark dataset. The key to doing this is to use the palindrome peptide FGAIAGF as the maker to divide all H1–16 sequences into HA1 and HA2. Coincidentally, peptide FGAIAGF has already been used earlier as a marker to divide HA1 and HA2 ([27], Skehel and Waterfield 1971).
2. Rebuild HA1A and HA1B based on the datasets of HA1. The key to doing this is to use the highly conserved peptide WGIHHP as the marker found by MCABMSA with *r  = * 0.001 to align the dataset of HA1.
3. Rebuild HA2A and HA2B based on the datasets of HA2. The key to doing this is to use the highly conserved peptide YNAELLV as the marker found by MCABMSA with *r  = * 0.001 to align the dataset of HA2.
4. HA1B is a longer segment and all amino acids on the head of a spike are contained in the first region of this segment. Using the peptide C***C****G which is located at the head of a spike but links the stem of the spike as a marker, we can further divide HA1B into HA1B1 and HA1B2.
5. HA1A, HA1B2, HA2A and HA2B are four segments containing the regions 1–58 and 302–344 on HA1 and the regions 3–87 and 94–196 on HA2, respectively.

These four regions form the stem of HA. For more details regarding how to divide the stem and head the reader is referred to Section 6 of Information S1. The details on how to get datasets of HA1A, HA1B2, HA2A and HA2B are shown in Section 7 of Information S1. Based on the datasets of HA1A, HA1B2, HA2A and HA2B, we can reanalyze the conservation of peptides in four regions site-by-site in five-group fashion. The final result is shown in Tables S13 and S14 separately in Section 8 of Information S1. Comparing Table S12 with Tables S13 and S14, we find that our conservation analysis is just an extension of Table S12 used in ref. [2].

**Figure 1:**
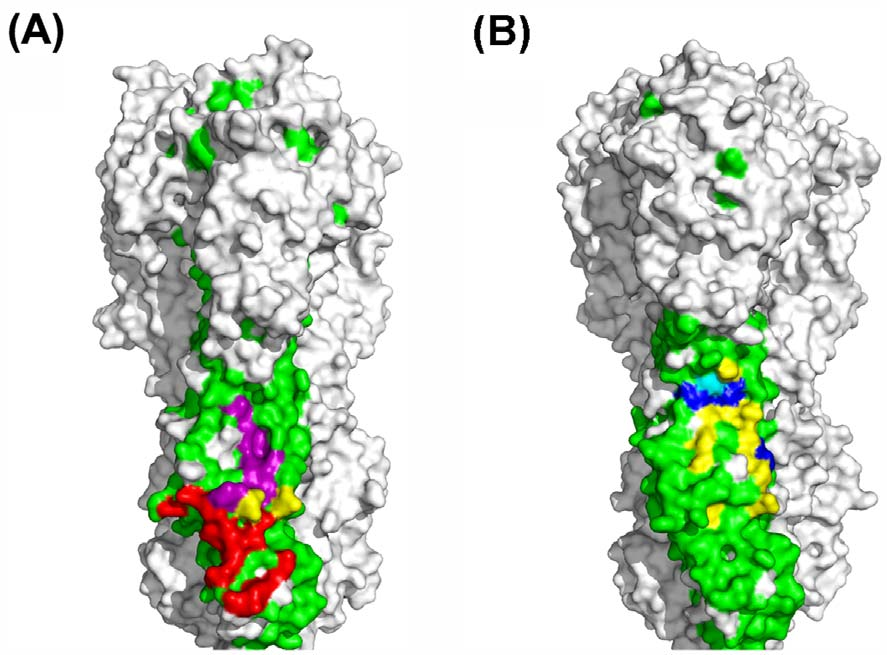
Comparison between the conserved area and the footprints. (A) The conserved area (green), the footprint of CR8020 (red), and the footprint of FI6 (purple) on group2 HA. The overlap between footprint of CR8020 and footprint of FI6 is yellow. (B) The conserved area (green), the footprint of CR6261 is Cambridge blue and yellow, the footprint of F10 is navy blue and yellow, and the footprint of FI6 is completely covered by yellow on group 1 HA.

Using 1rd8 and 1 mql as the models of group 1 HAs and group 2 HAs, we read off the common conserved area on group 1 HAs and group 2 Has, respectively based on Tables S13 and S14. Furthermore, we find the footprint of CR6261 on H1 HA, the footprint of F10 on H5 HA and the footprint of FI6 on 1rd8, respectively. For clarity, we highlight the conserved area with yellow, the footprint of CR6261 with red, the footprint of F10 with blue, and the footprint of FI6 with green. In the same way, the footprint of CR8020 on H3 and the footprint of FI6 on H3 are found on 1 mql, respectively. Shown in yellow is a conserved area, the red area means the footprint of CR8020 and the green area means the footprint of FI6. This is shown in Figure 1 (A) and (B).

Figure 1 (A) shows that the conserved area on group1 almost contains the footprints of CR6261, F10 and FI6, and all footprints contain the epitope (fusion peptide and the 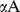
helix). Figure 1 (B) shows that the conserved area on group 2 almost contains the footprints of CR8020 and FI6. Notably, the footprint of CR8020 does not contain the epitope, which means that it is important to review the role of mAb. That is, the location of the footprint is not the key, but how to prevent HA from being separated is the key. Therefore, how to improve the affinity between mAb and HA and how to prevent the structure of a trimer HA from being broken is crucial.

In Figure 1 (A) and (B) we also find a green line over the union of all footprints, which corresponds to the peptide C***C****G*****PFQN. Moreover, the highly conserved peptides GECPKYV and LRLATGLRNVP are also outside of the union of all footprints. This also provides important side information. The asterisk * means that the amino acid of H1 HA and the amino acid of H3 HA in this position are different but it is almost invariant within H1 or H3 taken individually. Specifically, C***C****G (277–286) is located in the head of HA, while PFQN (sometimes PFHN) is located at the stem of HA.

### Determining the Molecular Mechanism of Action

#### The exploration of the binding pocket using a panel of drugs

Since Oseltamivir and Zanamivir may enhance the therapeutic efficacy of CR6261 to treat group 1 influenza viruses, we infer that 3 gbn and 3 gbm must be the target proteins for Oseltamivir and Zanamivir. To explore where on 3 gbn or 3 gbm the binding pocket of Oseltamivir and Zanamivir is located, we use AutoDock to search for the pocket using computational methods because we have no experimental means to do so. How to use it with a panel of drugs and a panel of target proteins to enhance the reliability of AutoDock has been mentioned in Section 1.2 of the Introduction. Typically, we explore the location of the binding pocket as follows.

1. Based on the panel of drugs and the panel of target proteins, we use AutoDock to blindly dock, one by one, the 9 drugs with the 6 target proteins.
2. Within the output of the drug and protein pair, we choose the pose ranked number 1 in the first cluster to be the optimal pose for each drug and protein pair.
3. On each target protein, we use PyMOL to show all of these optimal poses with negative values of MFE at the same time.
4. The cave/groove is called a binding pocket if it contains the union of these optimal poses with a negative value of MFE.

**Figure 2:**
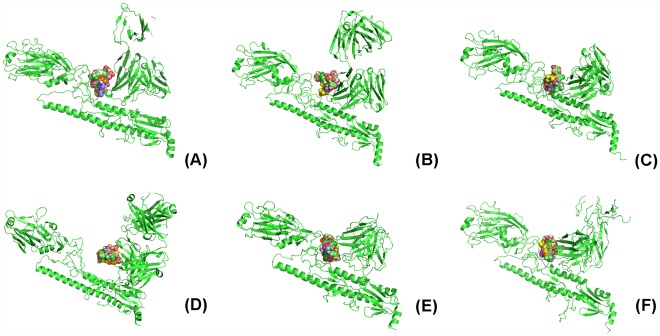
The pocket on 3 gbn, 3 gbm, 3 fku, 3 sdy, 3 ztn and 3 ztj explored using AutoDock based on one subunit. (A) The pocket of drugs on 3 gbn is the fork. (B) The pocket of drugs on 3 gbm is the fork. (C) The pocket of drugs on 3 fku is the fork. (D) The pocket of drugs on 3 sdy is the fork. (E) The pocket of drugs on 3 tzn is the fork. (F) The pocket of drugs on 3 tzj is the fork.

Using the above method of exploration, we obtain six pockets shown in Figure 2 (A)–(F) which represents the cleft formed by mAbs and HAs. We denote them as pocket_3 gbn, pocket_3 gbm, pocket_3 fku, pocket_3 sdy, pocket_3 ztj and pocket_3 ztn, respectively. Figure 2 (A)–(B) shows that the angles formed by H1 HA and CR6261, H5 HA and CR6261 (i.e., 3 gbn and 3 gbm) are different even though the footprints are the same. Figure 2 (E)–(F) (i.e., 3 ztn and 3 ztj), shows that both the angles and footprints of FI6 on group 1 and group 2 HAs are different.

Let the pose of the drug bound to the pocket with the globally minimum free energy be the optimal pose. Then slight differences between the angles will result in variations of the optimal pose. Intuitively, Figure 1 (A) shows that the optimal pose of Oseltamivir docked to pocket_3 gbn can improve the affinity between H1 HA and CR6261 because that Oseltamivir links HA with CR6261, while Figure 1 (B) shows that the optimal pose of Oseltamivir docked to pocket_3 gbm will not do so because Oseltamivir only binds to CR6261.

In order to quantitatively analyze the additional affinity provided by a drug in the optimal pose, we use Ligand Explorer to compute all hydrogen bonds and all hydrophobic interactions for each optimal pose. For simplicity, we do not distinguish hydrogen bonds and hydrophobic interactions and both are called by a joint name, non-covalent bonds. Then all non-covalent bonds can be classified into two sets. The non-covalent bonds between the ligand and HA are classified into the left set and the non-covalent bonds between the ligand and mAb are classified into the right set. We denote the numbers of left and right sets by a, b respectively. It is clear that a+b is proportional to the total binding affinity of a drug docked to the given pocket in the optimal pose. It is logical to expect that min{a, b} rather than a+b is the key parameter for improving the affinity between HA and mAb. Therefore, we infer that the therapeutic efficacy of CR6261 will be improved by a drug if min{a, b}>0.

Specifically, for the 9 optimal poses produced by the 9 drugs docked to pocket_3 gbn, we find the values a, b, a+b and min{a, b} for the 9 optimal poses on pocket_3 gbn, which are shown in Table 3. This shows that Oseltamivir and Zanamivir hold promise to enhance the therapeutic efficacy of CR6261 for H1 influenza A viruses because min{a, b} = 4. Aspirin and Heroin appear to be ineffective since min{a, b} = 0. Isosorbide and Amantadine also seem ineffective because min{a, b} = 1 is too small. Azithromycin is the best candidate among these 9 drugs since min{a,b} = 13 is the largest value found. This indicates that we may have found a molecular mechanism which explains why Oseltamivir/Zanamivir improves the therapeutic efficacy of CR6261 on H1 influenza viruses.

**Table 3:** The distribution of non-covalent bonds of the drug on 3 gbn.

	H1	H5	H3
CR6261	**665**–**719**	**618–633**	*98–111
CR8020	*126–136	–	**490–534**
F10	–	**654–688**	*170–180
FI6	**838–922**	–	**481–502**

Here, a is the number of non-covalent bonds between a drug and HA; b is the number of non-covalent bonds between a drug and CR6261; a+b is the total number of non-covalent bonds between a drug and the complex HA and CR6261; and min{a, b} is the minimum number between a and b. This table shows that Azithromcyin is best overall; Oseltamivir and Zanamivir also enhance the prophylactic and therapeutic efficacy of the CR626 antibody. Aspirin and Heroin appear to be ineffective. Both Isosorbide and Amantadine are almost ineffective.

We further compute the values a, b, a+b and min{a, b} of the 9 optimal poses for pocket_3 gbm, pocket_3 ztn, pocket_3 ztj, pocket_3 fku and pocket_3 sdy, respectively. The results are shown in Table S16 of Section 9 of Information S1. Contrary to our expectation, we find that min{a, b} = 0 in pocket_3 sdy for all 9 drugs, and min{a, b} = 0 for Oseltamivir and Zanamivir in pocket_3 gbm. The latter result can be interpreted as an inability of Oseltamivir and Zanamivir to improve the therapeutic efficacy of CR6261 for H5 if this binding site is unique in this pocket. However, this is contrary to the results obtained in the experiment of Friesen et al. This contradiction causes us to rethink the other poses of Oseltamivir within the pocket_3 gbm.

For the simplicity of computation, we will not distinguish between the pose and the coordinates. This is because each pose is determined by recalculated coordinates of the drug obtained through shifting or rotating its original 3D coordinates. Conversely, renewed 3D coordinates of the drug also uniquely determine a pose.

#### The neighborhood of a pose

A minor flaw of AutoDock which has been mentioned in Section 1 of Information S1 is that the optimal pose of Indinavir is not same as its real pose on the HIV-1 protease. Huey et al in 2007 [11] ascertained that the root-mean-square deviation (RMSD) between the real pose and the optimal pose is approximately 2.5 angstroms. For a given pose, we define the set of these poses within the pocket, which are within a 2.5A distance of the given pose, as the neighborhood of the pose. Clearly, the number of the poses in the neighborhood of a given pose is infinite. Therefore, this will also lead to a huge number of associated poses if we move the optimal pose to a real pose within the neighborhood of the optimal pose by applying operation of shifting and rotating. In other words, in practice we cannot compute all distributions of the non-covalent bonds for all associated poses. It is fortunate that distributions of non-covalent bonds are invariant if two associated poses are close enough. Therefore, due to the compactness of the neighborhood of a pose we may need to check a finite number of associated poses rather than to check all possible associated poses within the neighborhood.

It is not hard to imagine that a drug docked to a benchmark pocket will exhibit multiplicity of conformers (multiformity) because there is no means to ensure it must exactly bind to its benchmark pocket according to the designed pose. In fact, based on the available experimental results measured by x-ray crystallography, we can explore this multiformity of experimentally determined poses. For example, ADP is a well-known ligand which binds to many hundreds of proteins and has many benchmark pockets on each target protein. For example, the first group contains 12 proteins which are 1×88_A, 1 yrs_A, 2 fky_B, 2fl2_A, 2fl6_A, 2g1q_B, 2q2y_A, 2q2z_B, 2 wog_A, 2×7d_A, 2×7e_B and 3 cjo_A. The second group contains 11 proteins which are 1 bmf_D, 1e1q_D, 1e1r_D, 1efr_D, 2ck3_D, 2jiz_D, 2jj1_D, 2jj2_K, 2v7q_F, 2w6e_D and 2 wss_M. All of these proteins may be bonded by ADP. We assume that 12 poses are the poses of ADP docked to the same protein 1×88_A by independently repeating it 12 times and that 11 poses are the poses of ADP docked to the same protein 1 bmf_D by repeating it independently 11 times. Gathering the 12 poses on 1×88_A and the 11 poses on 1 bmf_D, we find that in practice ADP docks to the same benchmark pocket on 1×88_A or 1 bmf_D with multiple poses. A detailed validation is stated in Section 10 of Information S1.

Generally, it is believed that the molecules of a drug randomly collide with the benchmark pocket with no control over the exact docking mode to the benchmark pocket. Therefore, the real binding poses span a manifold within a neighborhood of a given pose. A drug is effective if most of the actual poses play the same effective role in binding to the target. Conversely, a pose is ineffective if all poses in the neighborhood of the pose play the same ineffective role.

For convenience, we define the quotient of these associated poses in the neighbor of the given pose playing the same role as the given pose versus the total number of poses in the neighbor as the tolerance of the pose. Following from the definition of tolerance, we easily find that a drug has high efficacy if the designed pose of the drug binding to its benchmark pocket has high tolerance. As mentioned before, the number of associated poses within the neighborhood of a given pose is huge. Therefore, the computation of the tolerance of a pose in practice should be approximated by a simplified method. For n associated poses, if m associated poses play the same role as the given pose, then we use the ratio m/n as the estimation of the tolerance of the given pose.

Specifically, for the optimal pose of Oseltamivir or Zanamivir binding to the pocket_3 gbm, we have randomly selected many associated poses in the neighborhood of the optimal pose and computed the numbers a and b and we find that min{a, b} = 0 for all of these selected associated poses. Therefore, the optimal pose of Oseltamivir or Zanamivir binding to the pocket_3 gbm is ineffective. This unexpected result further leads us to look for the real cause that Oseltamivir or Zanamivir improves the therapeutic efficacy of CR6261 to treat H5 influenza viruses.

#### The size of the benchmark pocket

Analyzing the size of pocket_3 gbm, we find it is large enough to be filled with more than two molecules of Oseltamivir. Therefore, we classify pockets into two classes according to a given drug. For a given drug, we say the pocket is small relative to the drug if the pocket cannot be filled with two or more molecules of the drug. Otherwise, we say that it is a large pocket relative to the drug. In other words, a small pocket may only have one neighborhood of the optimal pose, while a large pocket may contain at least two neighborhoods of two poses without an overlap. For small pockets, we frequently do not find an associated pose in the neighborhood of the optimal pose such that min{a1, b1}> min{a, b} through shifting and rotating the optimal pose. However, for a large pocket, we frequently find an associated pose so that min{a1, b1} > min{a*, b*} and that the neighborhoods of these two poses do not overlap. Here, min{a1, b1} is the contribution of the associated pose and min{a*, b*} is the contribution of the optimal pose.

Specifically, the existence of an associated pose in a large pocket such that min{a1, b1}> min{a, b} for all 6 pockets and 9 drugs is shown in Table S17 of Section 9 of Information S1. In Table S17, for all 54 optimal poses we find 54 associated poses such that min{a1, b1} > min{a*, b*}. From the data in Table S17, we find that Oseltamivir and Zanamivir at an associated pose which is far from the optimal pose can substantially improve the affinities between CR6261 and group 1 HAs, between F10 and group 1 HAs, between CR8020 and group 2 HAs, and between FI6 and HA for all subtypes.

It appears that the essential cause that Oseltamivir and Zanamivir may improve the therapeutic efficacy of CR6261 to treat group 1 influenza viruses has finally been found. However, there is still a gap that should be closed. That is, we need to answer whether the drug’s molecule may arrive in the neighborhood of the associated pose? We answer the question by stating the following property:

If a drug molecule in a large pocket has two poses such that their neighborhoods do not overlap, then the neighboring pose may be adopted by the drug molecule.

The proof of this property is not hard to arrive at. In fact, we regard a pocket just as the union of the two neighborhoods. Then this pocket is regarded as a bag with two boxes and each box can be packed into one ball. Since more than two molecules of the drug may enter into the pocket, it implies that more than two balls may enter into the bag. Therefore, it is certain that each box binds one ball.

#### The molecular mechanism to enhance the therapeutic efficacy of mAb

Based on the above computational analysis, we summarize the clues found and propose the molecular mechanism of action as follows:

1. For CR6261 (F10) which may neutralize group 1 influenza viruses and their footprints which contain the epitope, a drug may enhance the therapeutic efficacy of CR6261 (F10) if and only if it has a high tolerance pose satisfying min{a, b}>0 for group 1 HAs.
2. For CR8020 which may neutralize group 2 influenza viruses but its footprint is located on HA2 although it does not contain the epitope, a drug may enhance the therapeutic efficacy of CR8020 if and only if it has a high tolerance pose satisfying min{a, b}>0 for group 2 HAs.
3. For F16 which may neutralize both group 1 and group 2 influenza viruses and their footprints contain the epitope, a drug may enhance the therapeutic efficacy of FI6 if and only if it has a high tolerance pose satisfying min{a, b}>0 for all HAs.

Following from the above mechanisms and using the data in Table S17, we deduce that both Oseltamivir and Zanamivir may enhance the therapeutic efficacy of CR6261, CR8020, F10 and FI6 within their original spectrum. The other drugs do not lead to stable associations with Oseltamivir and Zanamivir having a high tolerance pose.

### The Drug Resistance of Antibody-drug Complexes

To identify an antibody-drug complex without drug resistance, we should estimate the affinity between HA and mAb. We should also determine how much affinity would be lost if the HA is changed and how much additional affinity would be provided by the drug. Moreover, it is useful to know whether or not the added affinity depends on the specific HA. We answer these questions one by one in the subsection that follows.

#### Estimation of the range of affinity between an antibody and HA

The affinity between an mAb and an HA is determined by multiple factors (i.e., hydrogen bonds, hydrophobic interactions, van de Waals forces, etc.), therefore we can hardly compute its value precisely. In practice, we use GROMACS to directly estimate the affinity between antibodies and HAs based on 3 gbn, 3 gbm, 3 fku, 3 sdy, 3 ztn and 3 ztj. Typically, we may use a large constant force (2,500 kJ/mol nm) to pull the CR6261, CR8020, F10 and FI6 far away from an HA along a fixed direction. We plot the distance-versus-time function and look for the time interval corresponding to a distance of 1A. We then trace it back to find the output energies corresponding to the given time interval. We use the energy corresponding to the left terminal of the interval and the energy corresponding to the right terminal of the interval as the lower and upper estimates of the affinity between HAs and mAbs, respectively. Typically, we estimate the range of affinities between HAs and mAbs as shown in Table 4. From Table 4, we find that affinity depends on the subtype. This clue is vital, but we need to confirm it further.

**Table 4:** Estimates of the affinity between an antibody and HAs at 1A (in units of kJ/mol).

mAb	HAs	Potential contributed amino acids
CR6261	H1	A_38_H A_40_V A_41_N A_42_L A_291_S A_292_L; B_19_D B_20_G B_21_W B_38_Q B_41_T B_42_Q B_45_I B_46_D B_49_T B_52_V B_53_N B_56_I
CR6261	H5	A_38_H A_40_Q A_41_D A_42_I A_291_S A_292_M A_293_P A_318_T; B_19_D B_20_G B_21_W B_38_C B_41_T B_42_Q B_45_I B_46_D B_49_T B_52_V B_53_N
F10	H5	A_32_H A_34_Q A_292_S; B_18_V B_19_D B_20_G B_21_W B_38_K B_41_T B_42_Q B_45_I B_49_T B_52_V B_53_N
CR8020	H3	A_21_P A_325_E; B_15_E B_16_G B_17_M B_18_I B_19_D B_25_R B_26_H B_30_E B_31_G B_32_T B_33_G B_34_Q B_35_A B_36_A B_38_L B_146_N B_150_E B_153_R
FI6	H1	A_28_H A_29_S A_289_S A_316_T; B_18_V B_19_D B_20_G B_21_W B_38_L B_39_K B_41_T B_42_Q B_43_N B_45_I B_46_D B_49_T B_53_N B_56_I B_57_E
FI6	H3	A_38_N A_277_C A_278_I A_318_T; B_18_I B_19_D B_20_G B_21_W B_38_L B_39_K B_41_T B_42_Q B_43_A B_45_I B_46_D B_48_I B_49_N B_53_N B_56_I B_57_E

Here, the boldfaced data are estimated based on the real crystal structures, and the data marked with a star are estimated based on the predicted structures. The symbol “-” means that structures corresponding to the trimer HA and mAb are absent.

#### The maximal losing of affinity between mAb and HA as the HA changed

To estimate the maximum loss of affinity between CR6261 (F10, FI6) and HA as HA ranges for group 1 or the maximum loss of affinity between CR8020 (FI6) and HA as HA ranges for group 2, we alternatively estimate the maximum loss of the non-covalent bonds between CR6261 (F10, FI6) and HA in group 1 or between CR8020 (FI6) and HA in group 2. Therefore, we should know which amino acids in the footprint of CR6261 (F10, CR8020, FI6) are the potential contributors towards non-covalent bonds. Since the lengths of the hydrogen bonds or hydrophobic interactions are less than 4 angstroms, we readily find the potential contributors within the footprint for each case. This is shown in Table 5.

**Table 5:** All potential contributors within a footprint for each mAb on three HAs.

Target	Left contributors on HA	Right contributors on mAb
3 gbn	289N, 290S	70T, 72D, 79Y
3 gbm	289N, 290S, 291S	23K, 75A, 79Y
3 fku	35D, 38S, 40K, 293S	75S, 76T
3 sdy	18H, 20V, 17M, 18I, 20G,	56T
3 ztn	286I, A287N, 288T, 289S	28T, 29F, 30S, 31T, 73N, 74S, 76N
3 ztj	55P, 278I, 280E, 288I, 289P, 290N	28T, 29F, 30S, 76N

Obviously, there is no strict obligation to contribute non-covalent bonds. We use Ligand Explorer to filter these potential contributors which give no actual contribution from the set if we regard it as a ligand. Based on the conservation analysis mentioned in Section 2.2, we know that the loss of non-covalent bonds is due to the mutations of these real contributors which come from HA1 (A-chain) because the contributors which come from HA2 (B-chain) are invariant. That is, we only care about the mutations of these contributors on HA1.

In particular, we select all actual contributors for 3 gbn, 3 gbm, 3 fku, 3 sdy, 3 ztj and 3 ztn, and obtain the sum of non-covalent bonds contributed by these contributors, and further infer the maximum loss of non-covalent bonds as follows:

1. The total number of non-covalent bonds between CR6261 and H1 is 36. 11 of 36 are contributed by 291S, 38H, 40V and 42L on HA1. Then, 3 of these 11 are contributed by the atoms on the backbone or by the first level of residues, and therefore the maximum loss is 8 if H1 HA is replaced by other group 1 HAs.
2. The total number of non-covalent bonds between CR6261 and H5 is also 36. But 7 of the 36 are contributed by 293P, 291S, 38H and 42L on HA1. Then 1 of these 7 is contributed by the backbone and therefore the maximum loss number is 6, if H5 HA is replaced by other group 1 HAs.
3. The total number of non-covalent bonds between F10 and H5 is 23, and only 2 non-covalent bonds are contributed by the amino acids 292S and 32H on HA1. Then, the maximum loss number is 1 if H5 is replaced by other group 1 HAs.
4. The total number of non-covalent bonds between CR8020 and H3 is 34. Only 1 non-covalent bond is contributed by the amino acid 325E on HA1. Then, the maximum loss number is at most 1 if H3 HA is replaced by other group 2 HAs.
5. The total number of non-covalent bonds between FI6 and H1 is 44, 12 of the 44 are contributed by 289S, 28H and 316T on HA1. Then, the maximum loss number of the non-covalent bonds is 7 if we assume that all group 1 HAs are mutated from H1. This is because 5 are contributed by the backbone or the first and second level of the side chains of amino acids.
6. The total number of non-covalent bonds between FI6 and H3 is 26; 1 of these 26 is contributed by 318T on HA1. We find that maximum loss number of the non-covalent bonds is at most 1 if H3 HA is replaced by other group 2 HAs.

Using the above observations, the real contributors and all potential contributors listed in Table 5, the footprints of CR6261, F10 and F10 all involve the peptide sequence C***C****G*****PFQN. The maximum loss is overestimated. In practice, the real loss is often much less than that.

#### The left and right footprints of Oseltamivir for the pose with high tolerance

Inspecting Table S17 again, we find that Oseltamivir may supplement more than 10 non-covalent bonds to improve the affinities of 3 gbn, 3 gbm, 3 fku, 3 sdy, 3 ztn and 3 ztj. We wish to known if the contributions of Oseltamivir are affected by mutations. We list the contributors in the left and right footprints of Oseltamivir based on the 6 crystal structures.

Following these sites in different proteins, we can easily find where they are located. From Table 6, we find that C***C****G*****PFQN is the source of the left contributors of Oseltamivir docked with the forks of 3 gbn, 3 gbm, 3 fku, 3 ztn and 3 ztj.

**Table 6:** The left and right contributors of Oseltamivir docked with 3 gbn, 3 gbm, 3 fku, 3 ztn and 3 ztj for a pose with high tolerance.

drug	A	B	L	min	drug	A	B	L	min
Azichromycin	**13**	**16**	**10**	**10**	HEM	10	7	7	7
Aspirin	8	3	6	3	Heroin	6	0	13	0
Amantadine	9	7	0	0	Isosorbide	11	0	7	0
Oseltamivir	3	7	0	0	Zanamivir	9	0	9	0

For the H1-H2-H5 and H8-H9-H12 subgroups, C***C****G*****PFQN is conserved and the common form is CDAKCQTPQGAINSSLPFQN. However, it is slightly mutated in H6, H11, H13 and H16. For most HAs in group 2, C***C****G*****PFQN becomes CNSECITPNGSSIPNDKPFQN. It is slightly changed in H4, H14, H7, H10 and H15. Thus, the left contributors of Oseltamivir are conserved for all four mAbs.

In conclusion, the results of Sections 2.2 and 2.3 tell us that Oseltamivir may enhance the therapeutic efficacy of CR6261, F10 and FI6 to treat group 1 influenza viruses without resulting in drug resistance, and it can also enhance the therapeutic efficacy of CR8020 and FI6 to treat group 2 influenza viruses without causing drug resistance.

### The Molecular Mechanism to Promote a Wide Spectrum mAb to Become Universal

#### Confirming that CR6261 and F10 may connect to H3 HA and CR8020 may connect to H1 HA

In order to prove that a drug may enhance CR6261 and F10 to become universal mAbs, we must first confirm that CR6261 and F10 may bind to group 2 HAs. Similarly, in order to prove that a drug may enhance CR8020 to make it a universal mAb, we must first confirm that CR8020 may bind to group 1 HAs. However, this is hard to do in the absence of crystal structures for these possible mAb-HA complexes. As the best recourse, we use RosettaDock to simulate how CR6261 and F10 bind to H3 HA and how CR8020 binds to H1 HA.

Of course, the precondition is whether we can use RosettaDock correctly. Therefore, we use 3 gbn, 3 gbm, 3 fku, 3 sty, 3 ztn and 3 ztj as samples to validate that RosettaDock has the ability to obtain similar results to those of the 6 crystal structures. Unfortunately, the results computed by RosettaDock are quite different from the known 3 gbn structure, if we first separate CR6261 and A-chain+B-chain from 3 gbn, and then input CR6261 and A-chain+B-chain into RosettaDock. In the same way, we find the computed results of RosettaDock for 3g bm, 3 fku, 3 sty, 3 ztn and 3 ztj are also quite different from their crystal structures.

**Figure 3:**
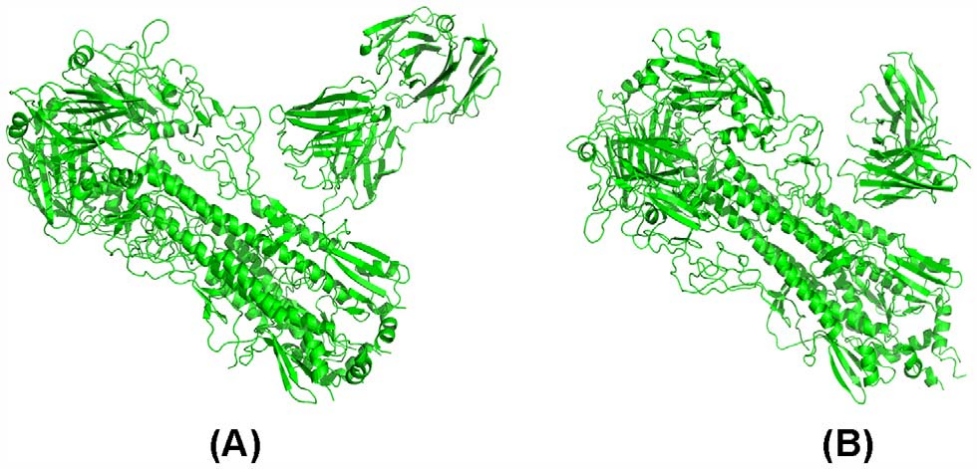
The validation of the reliability of RosettaDock. (A) The output of RosettaDock at the 338th iteration when the inputs are the trimer HA and FI6 which are separated from 3 ztj. (B) The output of RosettaDock at the 263^rd^ iteration when the inputs are the trimer HA and F10 which are separated from 3 fku.

Accidentally, we have found that 3 ztj has complete trimer HA data and we input the trimer HA and FI6 into RosettaDock. Then we use the docking pose with the minimum free energy within 1,000 iterations as the computed docking pose between the trimer HA and FI6. We find that the computed pose is very similar to the original crystal structure (see Figure 3(A)).

Encouraged by this finding, we continue our search for the trimer HA-mAb complex. Nevertheless, only for 3 fku (the H5 HA in complex with F10) can we find a complete trimer HA-mAb complex. In the same way we used to process 3 ztj, we obtain the computed pose of F10 docked with trimer HA and we compare it with the original crystal structure of 3 fku shown in Figure 3B. In other words, using mAb to dock to the trimer HA, RosettaDock has obtained a reliable pose.

**Figure 4:**
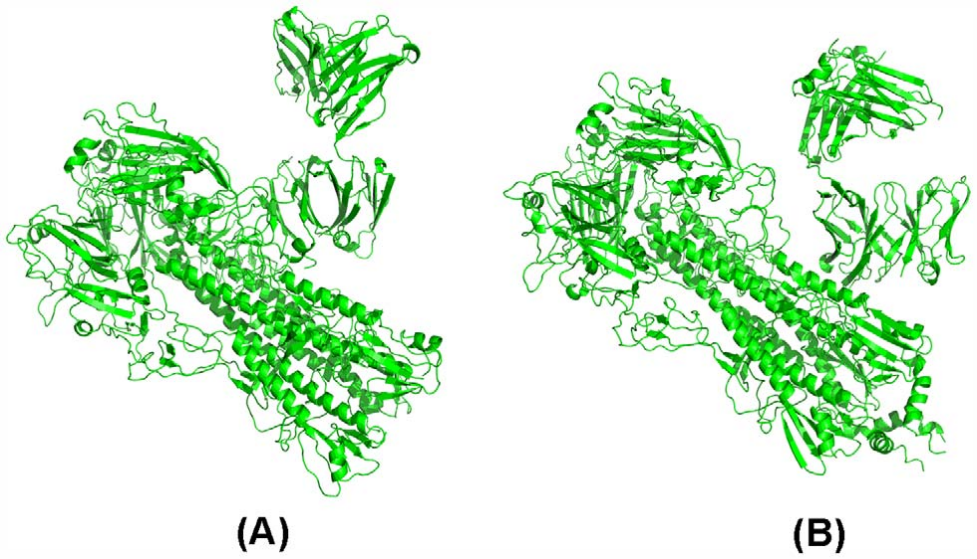
The validation of the reliability of RosettaDock. (A) The output of RosettaDock at the 200th iteration when the inputs are 1rd8 and CR6261. (B) The output of RosettaDock at the 363^rd^ iteration when the inputs are 2 ibx and CR6261.

In order to find a trimer HA based on 3 gbn and 3 gbm, we have to use the trimer 1rd8 to replace the trimer HAs corresponding to 3 gbn, and the trimer 2 ibx to replace the trimer corresponding to 3 gbm, respectively. We obtain a docked pose for 1rd8-CR6261 and a docked pose for 2ibx-CR6261 through RosettaDock shown in Figure 4 (A)–(B).

Based on Figures 3 and 4, we conclude that RosettaDock offers a real potential to capture the actual docked pose between a trimer HA and an mAb. We are confident that the trimer HA-mAb docked pose computed by RosettaDock is consistent with a global free energy minimum.

**Figure 5:**
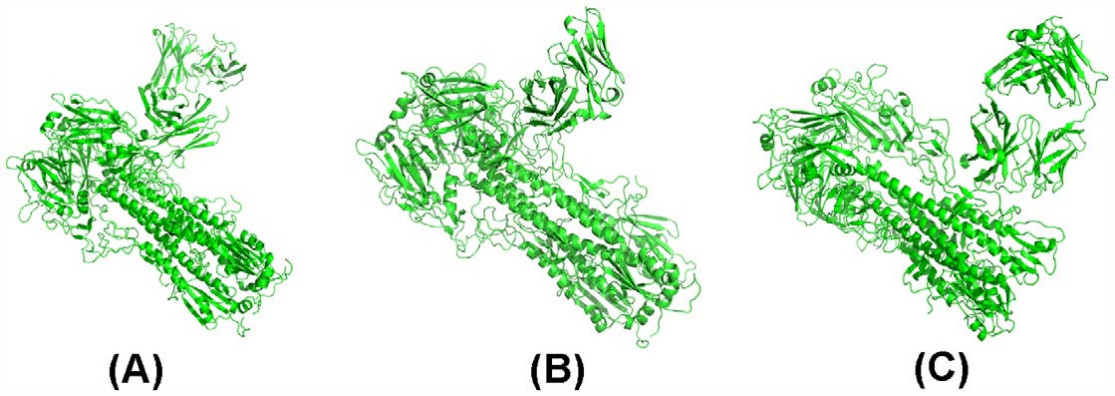
The predicted structures output obtained by RosettaDock: (A) is the predicted structure of CR6261 docking with H3 HA which is separated from 3 ztj, (B) is the predicted structure of F10 docked with H3 HA which is separated from 3 ztj, (C) is the predicted structure of CR8020 docked with 1rd8.

To check whether or not CR62661 (F10) may dock with group 2 HAs, we only show that CR6261 (F10) may dock with the trimer HA of H3 separated from 3 ztj. Similarly, to check whether or not CR8020 may dock with group 1 HAs, we only show that CR8020 may dock with 1rd8. With the same operation, we obtain the computed poses of CR6261 docked with H3 and F10 docked with H3 as shown in Figure 5 (A)–(B), and the computed poses of CR8020 docked with 1rd8 as shown in Figure 5 (C). Figure 5 (A)–(C), shows that the footprints of CR6261 and F10 are both located on the head of the spike of H3 HA and the footprint of CR8020 on H3 HA is located in the middle of the stem.

#### Confirming that drugs bind to the cleft formed by HA and mAb

**Figure 6:**
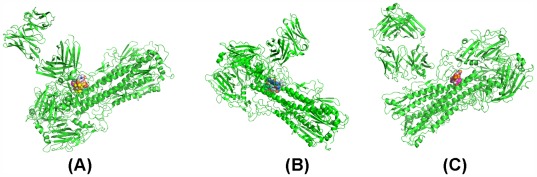
The pockets explored by AutoDock based on the complete predicted structures: (A) is the pocket on the predicted structure CR6261&H3, (B)is the pocket on the predicted structure F10&H3, (C) is the pocket on the predicted structure CR8020&H1.

Let CR6261&H3 denote the structure of Figure 5 (A), F10&H3 denote the structure of Figure 5 (B), and CR8020&H1 denote the structure of Figure 5 (C). We prove that a drug may bind to the fork formed by HA and mAb. However, an unexpected result has occurred. All drugs are not sent to the fork but to the trimer HA. We show the location of the pocket on CR6261&H3, F10&H3 and CR8020&H1 explored by AutoDock in Figure 6(A)–(C).

**Figure 7:**
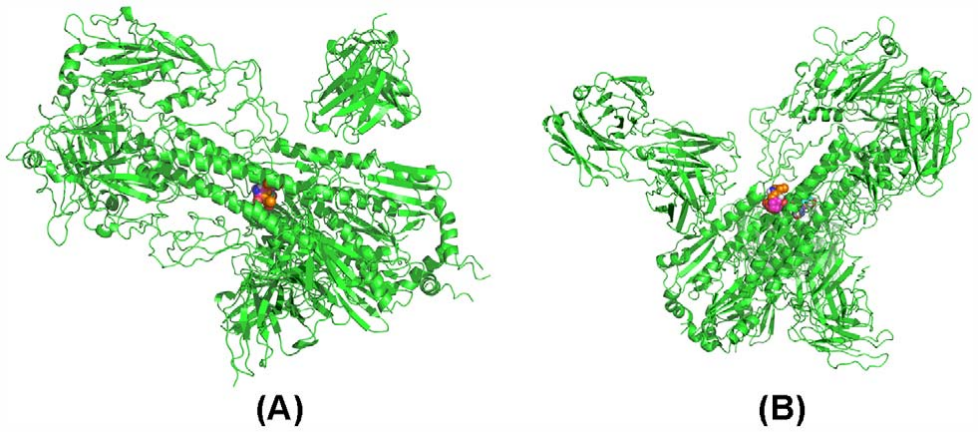
The pockets explored by AutoDock based on the complete real structures: (A) is the pocket on 3 fku, (B)is the pocket on 3 tzj.

At first, we suspected this result was wrong because CR6261&H3, F10&H3 and CR8020&H1 are computed structures. Nevertheless, we soon gave up this suspicion since we found that the drug does not come to the fork but to the stem of HA when we use the crystal structures 3 ztj and 3 fku (see Figure 7(A)–(B)).

This indicates that pockets on CR6261&H3, F10&H3 and CR8020&H1 explored by AutoDock are reasonable. Moreover, we further find that these pockets on the complete 3 fku complex, the complete 3 ztj complex, CR6261&H3, F10&H3 and CR8020&H1 explored by AutoDock are quite different from the pockets explored on the corresponding timer HAs. In fact, if we only use 1rd8, 1 mql, 3 m5 g and 2 ibx as models, then the pockets on each trimer HA are located almost in the same place (see Figures S17–S20 in Section 1 of Information S1; these pockets are very small and cannot be packed by Oselamivir or Zananmivir). That is, the pocket explored by AutoDock on the complete trimer HA-mAb complex is novel.

The above computational analysis tells us that a combined protein may have many pockets but these pockets should be explored using different subunits as the target proteins. In order to show that clefts formed by CR6261 and H3 HA, F10 and H3 HA, and CR8020 and H1 HA are pockets, we need to use a single strain complex with CR6261, F10 and CR8020 as target proteins. This is shown in Figure 7(A)–(C). Furthermore, we also find a pocket for drugs on mAb if we only use mAb as the target protein. The pockets of CR6261, CR8020, F10 and FI6 are shown in Figures S14–S16 in Section 1 of Information S1.

#### Osletamivir may promote CR8020 to be a universal mAb

We first note the structure of CR8020&H1, since the footprint of CR8020 on H1 HA is at the stem and it covers the epitope of HA. However, the number of non-covalent bonds between HA and CR8020 is only 4. Furthermore, using GROMACS, we also find that the affinity between H1 HA and CR8020 ranges from 126 to136 kJ/mol (see Table 4). Therefore, a drug may enhance CR8020 to become a universal mAb if and only if it provides enough additional affinity so that CR8020 does not unbind from H1 HA.

Based on the trinity symmetry of the HA-mAb complex, we know that CR8020 in complex with H1 HA has at least nine pockets, which are large enough to be bonded by Oseltamivir and Zanamivir. In fact, three of them locate on the three faces of the stem of trimer HA, three locate on the three forks and three on three identical mAbs. Following from the CR8020&H1 case, we find that Oseltamivir may help CR8020 to neutralize H1 influenza viruses using the molecular mechanism described in Section 2.3. It is important to note that the footprint of CR8020 on H1 HA is located on the stem.

**Figure 8:**
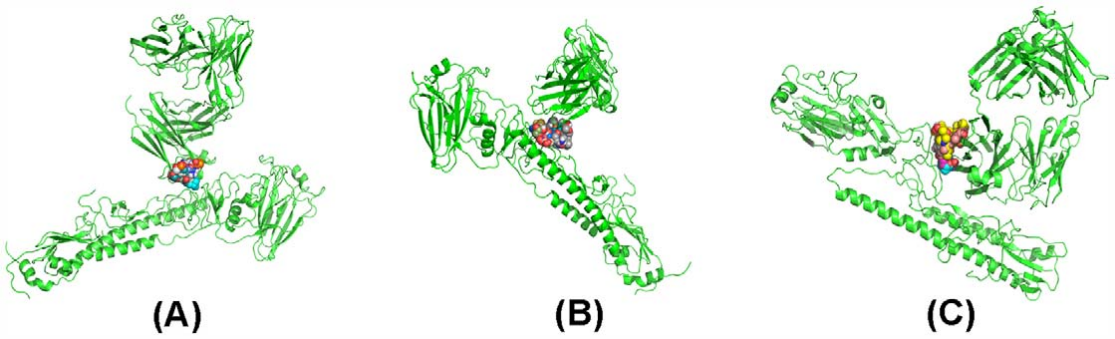
The pockets on the predicted structures explored by AutoDock based on a subunit: (A)is the pocket explored based on one subunit of the predicted structure CR6261&H3, (B)is the pocket explored based on one subunit of the predicted structure F10&H3, (C)is the pocket explored based on one subunit of the predicted structure CR8020&H1.

Following from the pose of Oseltamivir docked to the fork formed by CR8020 and HA shown in Figure 8C, we use Ligand Explorer to compute the distribution of non-covalent bonds. We obtain a = 10, b = 9 and min {a, b} = 9. Moreover, the associated poses produced by shifting the center of the coordinates in the optimal pose within 0.5A do not change the value of min{a, b}. Thus, we lean towards the conclusion that Oseltamivir may enhance CR8020 to become a universal mAb.

By comparison, the footprints of CR6261 and F10 on H3 HA both are located on the head of the HA and therefore can at most link to HA1 but cannot cause HA2 being separated from HA. In other words, a drug may enhance CR6261 or F10 to neutralize H3 influenza viruses if it prevents HA2 from dissociating from HA and if it provides additional affinity so that CR6261 or F10 may bind to the head of HA, too. Figure 6 (A)–(C) and Figure 8 (A)–(C) show that the pocket on CR6261&H3 or F10&H3 is different from the pocket on CR8020&H1. Therefore, the selection of drugs to enhance CR6261 or F10 as a universal mAb is more difficult. Moreover, based on a comparison of Figure 6A&Figure 8A with Figure 6B&Figure 8B, we further find that pockets on CR6261&H3 and F10&H3 are also different and therefore we need to discuss CR6261 and F10 separately.

#### Azithromycin may promote CR6261 to become a universal mAb

Figure 6(A) shows that that the footprint of CR6261 on H3 HA is on the head. Then using Ligand Explorer, we find only 11 non-covalent bonds between CR6261 and HA. Furthermore, we use GROMACS to estimate the affinity between CR6261 and HA which is found to be ranging from 98 to 111 kJ/mol. In other words, the affinity between CR6261 and HA is really weak. Moreover, the pocket of CR6261&H3 is very special. Two pockets are degenerated into one pocket relative to 3 ztj. Therefore, a drug that binds here must play two roles. One is to keep HA1 and HA2 closer together and the other is to keep CR6261 bound tightly to the head of H3 HA. It means that a drug may be selected as a candidate if and only if it binds to HA1 (A-chain), HA2 (B-chain) and CR6261 (L-chain) with a sufficient number of non-covalent bonds. Based on the optimal poses of the 8 drugs binding to the pocket, we have the distribution of non-covalent bonds as shown in Table 7.

**Table 7:** The numbers of non-covalent bonds distributed on three chains under the optimal poses.

Pocket on trimer HA-F10 complex				cleft formed by trimer and F10			
drug	B	D	F	drug	HA	F10	min
Azichromycin	0	0	0	Azichromycin	**18**	**15**	**15**
Aspirin	6	17	0	Aspirin	11	0	0
Amantadine	2	2	6	Amantadine	15	2	2
Oseltamivir	**21**	**10**	**9**	Oseltamivir	14	3	3
HEM	0	0	0	HEM	16	0	0
Heroin	**19**	**16**	**18**	Heroin	6	8	6
Isosorbide	8	6	0	Isosorbide	13	4	4
Zanamivir	15	4	8	Zanamivir	15	4	4
Vancomycin	0	0	0	Vancomycin	0	0	0

Following from Table 7, we find that Azithcromycin is the best candidate since the numbers of the non-covalent bonds between Azithromycin and HA1 (HA2, CR6261) are 13, 16 and 10, respectively. Moreover, the distribution of the non-covalent bonds is not changed significantly if the pose is replaced by its associated poses obtained by a slight shift since min{13, 16} = 13 is about 260 kJ/mol. Consider the three identical faces of the trimer HA. Then three molecules of Azithromcyin bind to the three identical pockets, which adds 780 kJ/mol of binding affinity between HA1 and HA2. This is large enough to make sure that HA1 is tightly linked to HA2. Moreover, the additional 10 non-covalent bonds may also improve the affinity between CR6261 and HA. Therefore, we believe that Azithcromycin is a good candidate to enhance CR6261 to become a universal mAb.

#### How to promote F10 to become a universal mAb

The footprint of F10 on H3 HA is also at the head. Moreover, the number of the non- covalent bonds between F10 and HA is also 11 as computed by Ligand Explorer. Furthermore, the affinity between F10 and HA estimated using GROMACS ranges from 170 to 180 kJ/mol (see Table 4). Figure 6B and 8B show that the selection of drugs for F10 is neither the same as the selection for CR6261 nor the selection for CR8020. In order to neutralize H3 influenza viruses, we should require the drug binding to the pocket on the trimer HA such that HA does not be break. Then, a drug molecule may bind to the exterior angle of the cleft formed by F10 and HA such that F10 does not unbind from HA. For this purpose we should compute the numbers of non-covalent bonds and how they are distributed in the corresponding pockets. We show these values in Table 8.

**Table 8:** The number of non-covalent bonds for each drug distributed on different parts.

ligand	a+b	a	b	Min{a,b}
Azithromcyin	36	23	13	13
Oseltamivir	17	13	4	4
Zanamivir	17	13	4	4
HEM	27	9	18	9
Aspirin	24	24	0	0
Isosorbide	21	20	1	1
Vancomycin	19	16	3	3
Amantadine	9	8	1	1
Heroin	18	18	0	0

Table 8 shows that a single drug cannot satisfy the requirement, and therefore we recommend that two drugs together combine to form a F10 –drug complex to enhance F10 to become a universal mAb. The left part of Table 8 shows that Oseltamivir is a good candidate to link the stem of the timer HA which does not become broken since the numbers of non-covalent bonds between Oseltamivir and B-chain (HA2), between Oseltamivir and D-chain (HA2), and between Oseltamivir and F-chain (HA2) are 21, 10 and 9, respectively. This strong affinity ensures that the B-, D- and F-chains do not fall to pieces. The left part of Table 8 also shows that Azichromycin does not bind to the pocket on the trimer HA and therefore it does not compete with Oseltamivir to occupy this pocket. The right part of Table 8 shows that Azithromycin has the strongest ability to fill the exterior angle of the cleft formed by F10 and HA and may provide 15 non- covalent bonds to improve the affinity between F10 and the stem of HA. Therefore, a possible conclusion stemming from this analysis is to recommend that Azithromycin and Oseltamivir are used together to promote F10 to neutralize H3 influenza viruses and so that F10 becomes a universal mAb.

## Results and Discussion

Summarizing the computational analysis in Sections 2.2 and 2.3, we have obtained the following results. We found a molecular mechanism that explains why Oseltamivir and Zanamivir may enhance the therapeutic efficacy of CR6261 for treating group 1 influenza viruses. Based on this mechanism, we further found that Oseltamivir and Zanamivir may also enhance the therapeutic efficacy of F10 or FI6 for treating group 1 influenza viruses and the therapeutic efficacy of CR8020 or FI6 for treating group 2 influenza viruses. Moreover, these drugs may compensate for the loss of affinity between HA and mAb due to some mutations of amino acids within their footprints.

Using the RosettaDock software, we found that the footprint of CR8020 on H1 HA is located in the middle of the stem of HA which is better than the footprint of CR8020 on H3 HA. However, the affinity between CR8020 and H1 HA is too low to keep CR8020 and H1 HA together sufficiently tightly (see Table 4). Therefore, we believe that a molecular mechanism to promote CR8020 to become a universal mAb has been found. We only need to identify a drug to enhance the affinity between CR8020 and the trimer HA so that CR8020 cannot unbind from HA. Among the panel of 9 drugs examined, we found that Oseltamivir squeezes through the selection process. It is possible that a better drug can be found in the future using DrugBank or other chemical databases.

Using the RosettaDock software, we found that the footprint of CR8020 on H1 HA is located in the middle of the stem of HA which is better than its footprint on H3 HA. However, the affinity between CR8020 and H1 HA is too low to keep CR8020 and H1 HA bound sufficiently tightly together. Therefore, we believe we have found a molecular mechanism required in the search for a drug to enhance CR8020 to become a universal mAb.

Using the RosettaDock software, we found an explanation why CR6261 (F10) does not neutralize group 2 influenza viruses, namely we believe this is due to the fact that the footprints of CR6261 and F10 are located at head of the HA. Therefore, a molecular mechanism for using a drug to enhance CR6261 or F10 to become a universal mAb has been formulated. Based on the mechanism and distribution of non-covalent bonds between a drug and a binding pocket, we recommend Azichromycin as a candidate to enhance CR6261 to become an mAb. Moreover, we recommend Azichromycin and Oseltamivir together as a candidate to enhance F10 to become a universal mAb.

We hope that using DrugBank and other medicinal chemistry databases we will be able to find other drugs, which may play the same role as Oseltamivir, Zanamivir or Azichromycin. However, clinical experience shows that Azithromycin is safe (no reported lethal side effects yet). Its half-life is long (about 18 hours) and therefore it is convenient for clinical use. It causes no damage to human proteins unless bacteria residing in the human body are affected. Importantly, it is inexpensive. Hence, Azithromycin is a good candidate to help CR6261 and F10 become universal antibodies. Comparably, Oseltamivir has some side-effects and is much more expensive without offering an advantage over Azithromycin. Zanmivir is also a mature drug to treat influenza viruses aiming at the target protein NA. However, its mode of delivery is not convenient, although the price is not too high.

In summary, this study shows that Oseltamivir may improve the therapeutic efficacy of FI6 to overcome drug resistance. As well, Osetamivir may possibly enhance CR8020 to become a universal mAb. Azithromycin may enhance CR6261 to become a universal mAb and Azichromycin and Oseltamivir taken together may enhance F10 to become a universal mAb. Therefore, we have multiple choices to obtain cheap and safe antibody-based therapies. We should carefully note the order of drug delivery. The correct order should be that mAb docks with HA first, and then a drug should be added. Otherwise, the function of the mAb will be seriously lost. In fact, we have mentioned in Section 1 of Information S1 that there is a large pocket on CR6261, FI6, and CR8020. If we mix a drug and mAb first, then the drug will bind to mAb and the activity of mAb will be reduced. In fact, an experiment performed by a group at the Chinese CDC based on Sichuan SWL1/2009(CNIC)and California/7/2009(CNIC) shows that the therapeutic efficacy of the mixture of CR6261 and Azithromycin to treat group 1 influenza viruses would be half of the single CR6261.

Finally, we wish to comment on the use of MCABMSA software. Over the last three years we have validated it on many test datasets (general and specific) and compared it with MUSCLE and MAFFT. Regarding computational speed, MCABMSA is much fast than MUSCLE but almost the same as MAFFT. For the size of the dataset, MCABMSA is much larger than MAFFT. Regarding the SP-score, MCABMSA is superior to MUSCLE and MAFFT on average. Based on the study presented in this paper, MCABMSA emerges as having greater agility than the publicly available multiple sequence alignment packages. Readers may freely download it from http://mathbio.nankai.edu.cn/aligneddatabase.

Finally, we would like to emphasize that AutoDock, Ligand Explorer, GROMACS, and RosettaDock are four types of useful software packages that perform computational drug design and can reliably select antibody and drug complexes. We note that the speeds of AutoDock, Dock and GROMACS are much lower. If their speeds could be increased, they would be more useful in the applications aimed at finding new targets for old drugs. For the current cases studied here, we have to pay more attention to choosing the panel of drugs in order for them to span a wider spectrum of properties.

## Supporting Information

Information S1 **Supplementary material of the paper.**
(DOC)
