# Supplementary material for: The Molecular Mechanism of Action of the CR6261-Azichromycin Combination Found through Computational Analysis
Source: PLoS One. 2012 May 31;7(5):e37790. doi: 10.1371/journal.pone.0037790 (PMC3365099; doi:10.1371/journal.pone.0037790)
Supplement: Information S1 — Supplementary material of the paper. (DOC) [file pone.0037790.s001.doc]

Supplementary material for

**The molecular mechanism of action of the CR6261-Azichromycin combination found through computational analysis**

Wei Cui, Kui Wang, Jishou Ruan, Qi Zhi, Yi Feng, Yiming Shaoand Jack A. Tuszynski

College of Mathematical Sciences and LPMC, Nankai University, Tianjin, PRC 300071,

National Center for AIDS/STD Control and Prevention, Chinese Center for Disease Control and Prevention, Beijing, PRC 100800

Division of Experimental Oncology, Cross Cancer Institute, 11560 University Avenue, Edmonton, AB T6G 1Z2, Canada

State Key Laboratory for Medicinal Chemical Biology at Nankai University

*Corresponding author should be addressed: [jsruan@nakai.edu.cn](mailto:jsruan@nakai.edu.cn)

1. **The detail validation why all drugs may be predicted to be docked with same pocket**

Many years ago, we only process one ligand and one pocket at a time. When we studied the influenza viral drugs: Amantadine, Zanamivir and Oseltamivir, we downloaded 1nyj and 2hu4 from PDB since the target protein for Amantadine is 1nyj and the target protein for Zanamivir and Oseltamivir is 2hu4. Then we found out that Amantadine, Zanamivir and Oseltamivir can be docked into pocket_2hu4 and pocket_1nyj the same way but did not utilize it. When we compared the efficacies of Amantadine, Zanamivir and Oseltamivir with other non influenza viral drugs, we found that all drugs selected can be docked in the same neighbor of a pocket. As the number of the drugs increases, this law is unchanged. We were puzzled by the special phenomena. On one occasion, we suspected either we had misused the AutoDock or AutoDcok has some flaw because different drugs may be predicted to dock with the same place.

We first exclude that we had operated AutoDock wrongly because we had validated that Oseltamivir and Zanamivir can find their benchmark pocket on their target protein NA, that Amantadine can also find its benchmark pocket on its target protein M2, and that Fosamprenavir, Indinavir, Nelfinavir, Darunavir, Tipranavir and Amprenavir also find their benchmark pocket on their target protein HIV-1 protease.

We also exclude that AutoDcok has flaw after we validated this result on a large panel of proteins and a large panel of ligands. In fact, selecting 1rd8, 2hu4, 1nyj, 3cm8, 3hw3, 1g6l, 2jle, 2gv9, 3gbn, 3gbm, 3fku, 3sdy, 3ztn and 3ztj as the target proteins, and choosing Amantadine, Aspirin, Azithromycin, HEM, Heroin, Isosorbide, Oseltamivir, Zanamivir and Vancomycin as the panel of drugs, then all of these ligands are also predicted to be docked with the same pocket on each of above target proteins if these ligands can be packed into this pocket, while all of those ligands will arrive at the minimal value of minimal free energy at a neighbor of the pocket if those ligands can not be packed into this pocket. Moreover, when the panel of ligands is enlarged to 34 ligands (as shown below), these 34 drugs are also predicted to be docked with the same neighbor of a pocket uniformly.

Furthermore, for the proteins formed by subunits, if we just use a subunit as the target protein, then all ligands also are predicted to be docked with the same neighbor on the subunit of the pocket. Of course, this pocket on a subunit is not same as that pocket on entire protein. For example, using a subunit of 3hw3 and entire 3hw3, we have two pockets shown figure S1(A)-(B) respectively.


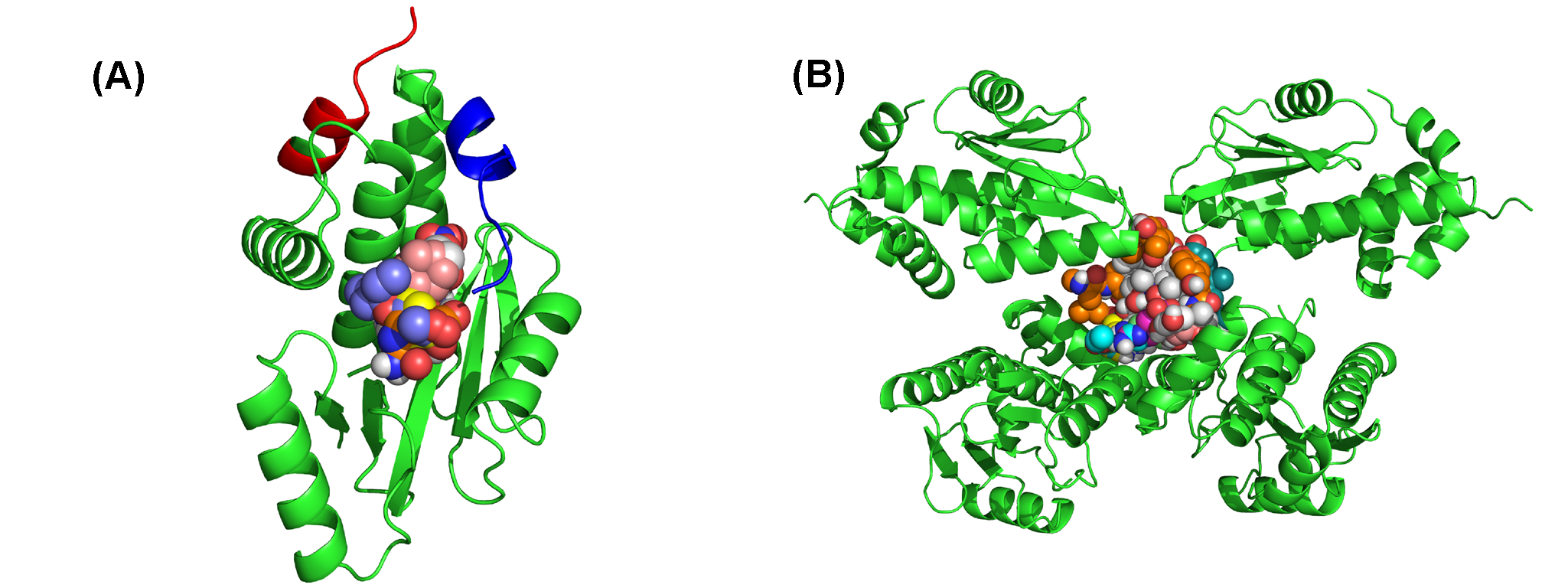


Figure S1. (A) Drugs bind to the position of metallic ions when a subunit of 3hw3 is the target protein. (B) Drugs bind to the void surrounded by four subunits when 3hw3 is the target protein.

Another example, we use the complex proteins formed by antibody and one subunit of the trimer HA (i.e., 3gbn, 3gbm, 3ztj, 3ztn, 3fku and 3sdy) as the target proteins, then the pockets explored by AutoDock are the fork formed by a subunit of HA and the mAb shown in figures S2 (A)-(F).


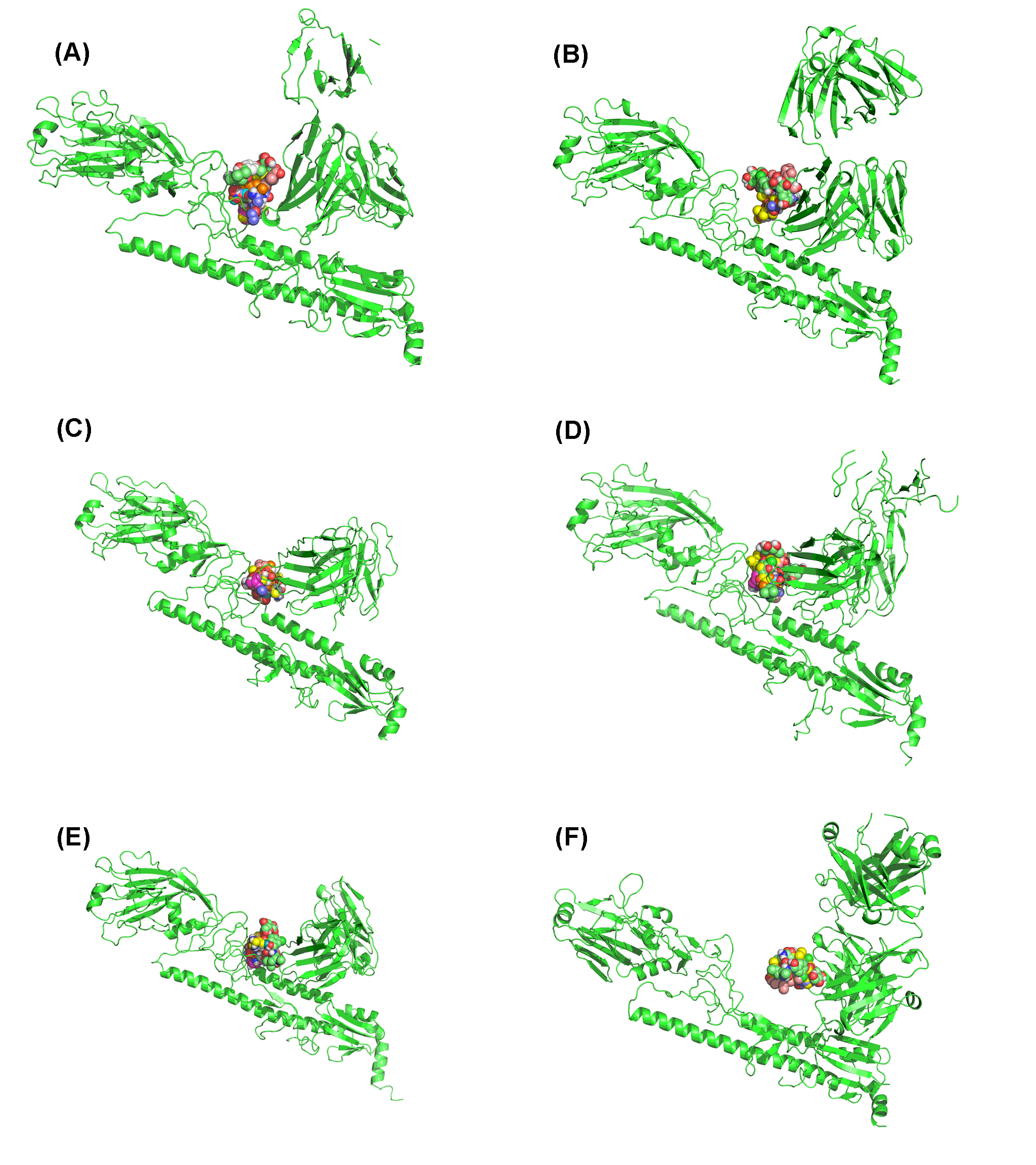


Figure S2. (A) Drugs docking with one subunit of 3gbn. (B) Drugs docking with one subunit of 3gbm. (C) Drugs docking with one subunit of 3tzn. (D) Drugs docking with one subunit of 3tzj. (E) Drugs docking with one sub unit of 3fku. (F) Drugs docking with one subunit of 3sdy.

However, if we use CR6261, F10, CR8020 and FI6 as the target proteins, then the pocket on each antibody is the blank formed by heavy and light chains from shown in figures S3(A)-(D)


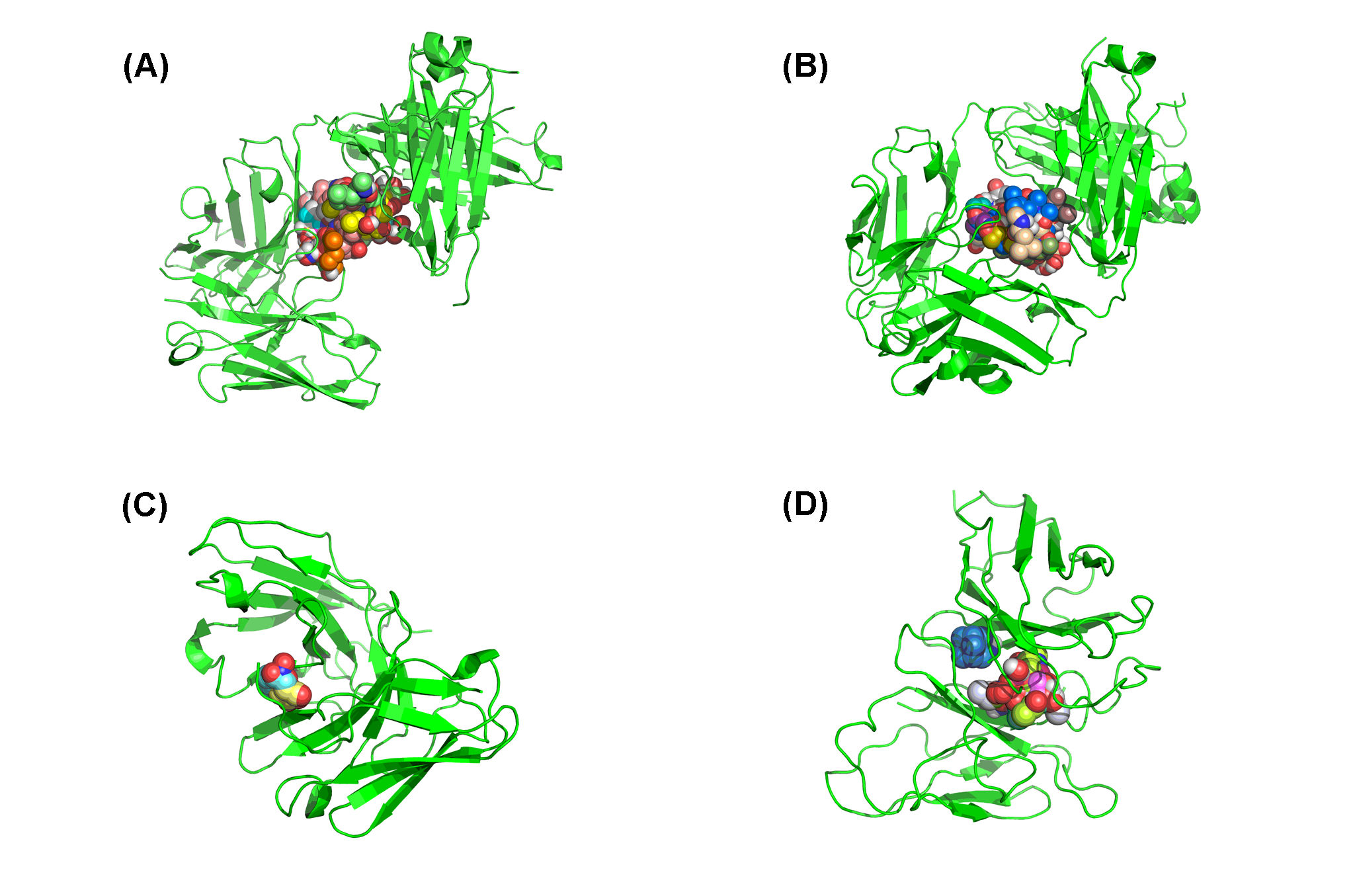


Figure S3. (A) Drugs docking with CR6261. (B) Drugs docking with CR8020. (C) Drugs docking with F10. (D) Drugs docking with FI6.

If we use H1 HA, H5 HA, H3 HA and H7 HA as the target proteins, then the pockets on each class are almost same shown in figures S4(A)-(D).


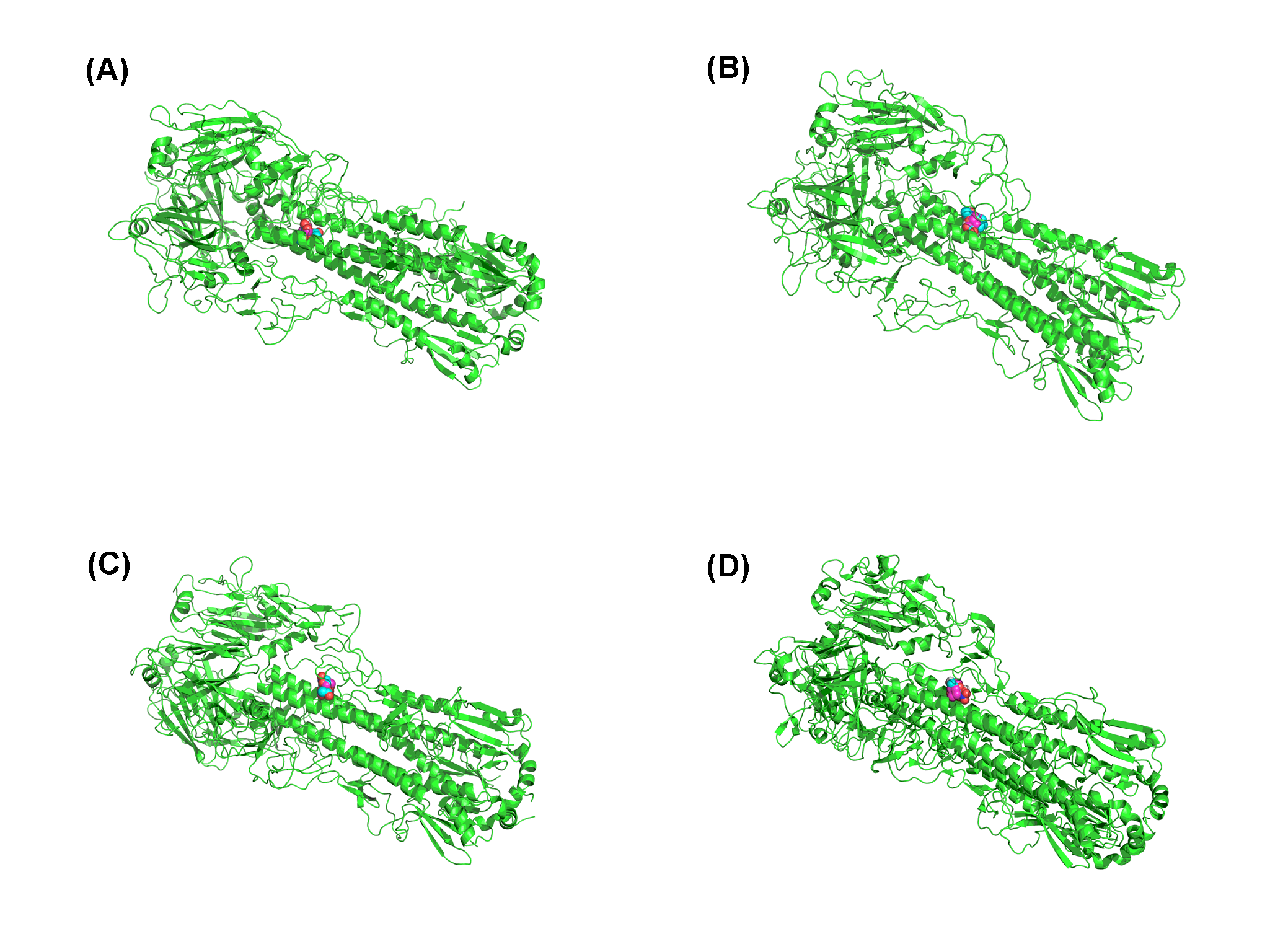


Figure S4. (A) The pocket on 1qml. (B) The pocket on 2ibx. (C) The pocket on 3m5g. (D) The pocket on 1rd8.

After above validation, not only we confidently trust that AutoDock is a reliable tool to find the pocket of drugs on the given target protein, but also believe that AutoDock must contain a good preprocessing subprogram so that AutoDock always may escape from the trap of the locally minimal value. In order to look for all potential pockets, we need used different subunit and entire unit respectively.

Encouraged by this advantage, we have the idea to utilize this advantage sufficiently. In fact, if we input the 3D coordinates of a drug and the 3D coordinates of a protein, then AutoDock will outputs a value of the minimal free energy (MFE) and a predicted coordinates of the drug. Also, if we input a panel of drugs with the 3D coordinates and the 3D coordinates of a protein, then AutoDock will output a series of values of MFE and the predicted coordinates of the drugs. Therefore, if we show out all of these drugs with negative MFE using PyMOL according to the predicted coordinates at same time, then these drugs will be clustered in a void or a groove. And then we say this void/groove on a given target protein is a benchmark pocket of these drugs.

Nevertheless, AutoDock still has a minor flaw. In practice, the predicted docking pose may not be perfectly same as the real pose observed using x-ray. For example, the pose of Indinavir docking with 2bpx (one type of HVI-1 protease) obtained through x-ray is not perfectly same as the pose of Indinavir docking with 2bpx predicted using Autodock, though both they are packed into the same benchmark pocket. See the figures S5(A)-(B).


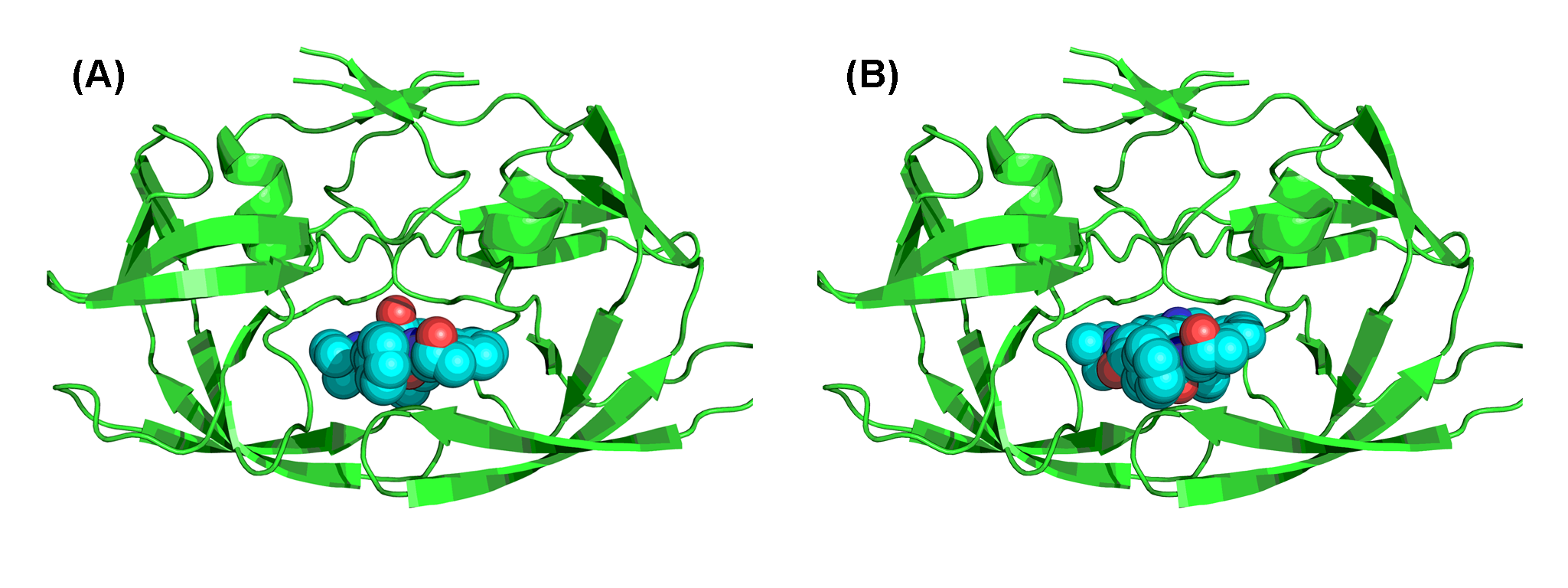


Figure S5. (A) The experimental effect of Indinavir binding to the pocket on 2bpx. (B) The docking effect of Indinavir binding to 2bpx using AutoDock.

Consequently, we should keep in mind that predicted pose of a dug docking with a pocket may not be perfect same as the real pose and the predicted pose is just a referential answer. In fact, we will use the experimental results shown in PDB database to prove that the real poses of a given drug docking with a given pocket may be too many.

1. **How to assemble the panel of drugs**

The panel of drugs is consisted of 9 drugs. Amantadine, Oseltamivir and Zanamivir are certainly selected because we know their benchmark pockets and their background in detail. However, the choice of the 6 non-flu drugs is really arbitrary. There is a long history related to this choice. While we are interested in drug design, we are not experts in pharmacology. On a personal note, one of the authors, Dr. JRuan, is a patient with heart trouble and he takes Aspirin and Isosorbine and some other drugs every day. Therefore, he knows from personal experience that these drugs are safe. We found they have smaller molecular weights and we checked they are included in DrugBank so we could determine the coordinates of the drugs. Azithromycin and Vancomycin are two popular antiphlogistic drugs, but we were not familiar with them before we studied influenza viruses. Heroin and HEM are included in the panel only for comparison purpose. Since we wanted to search for drugs liking Aspirin having acetyl groups, and found out that Heroin and Azithromycin are larger than aspirin and they have acetyls and methyl, they were therefore included in the study. Moreover, Heroin is a natural opioid anelgesic and Azithromycin is a popular antiphlogistic drug having low side-effects but long half life. Therefore, they were chosen for the panel. At first, we wanted to use the 7 drugs: Aspirin, Isosorbine, Herion, Amantadine, Zanamivir, Oseltamivir and Azichromcyin to form the panel to evaluate the efficacies of Amantadine, Zanamivir and Oseltamivir and we wanted the number of drugs in the panel to be odd. When we computed the sizes of these seven drugs we found the distribution of the sizes was too narrow. Since the size of Heroin is not significantly larger than the size of Oseltamivir, then only Azichromcyin’s size is greater than the size of Oseltamivir. To make the panel better balanced in size, we looked for two drugs with sizes bigger than the size of Oseltamivir. We found out that Vancomycin is larger and is an approved drug, thus we chose it for the panel. HEM is a natural product contained in DrugBank but is not an approved drug. Also, it is a larger ligand. Therefore, we included it in the panel. Then the final panel of 9 drugs is fair in size and contains a balance between positive samples and negative samples.

Luckily, according to the hint of the anonymous reviewers, we further search out the experimental target proteins of the 9 drugs from DrugBank, which may obviously tell us that 6 drugs we selected randomly are really non influenza viral drugs. And we summarize the basic knowledge of the 9 drugs in the table 1.

Table S1. The weight and the target protein of the drugs in the panel

| drug | weight | Target protein | state |
| --- | --- | --- | --- |
| Vancomycin | 1449.254 | 1pnv | approved |
| Azithromycin | 748.9845 | **50S ribosomal protein L4** | approved |
| HEM | 618.46 | 1bep | experimental |
| Heroin | 369.411 | **Mu-type opioid receptor** | illicit, experimental |
| Zanamivir | 332.3098 | NA (**Neuraminidase)** | approved |
| Ossltamivir | 312.4045 | NA (**Neuraminidase)** | approved |
| Isosorbide | 191.1388 | enzyme guanylate cyclase | approved |
| Aspirin | 180.1574 | COX-1/ COX-2 | approved |
| Amantadine | 151.2487 | Proton channel protein M2 | approved |

1. **How to obtain the HA Dataset**

Using the order influenza+HA, influenza+NA, etc, we can download the 11 datasets of HA, NA, M1, etc from the Uniprot database. Nevertheless, each dataset does not just collect the corresponding sequences. Taking the HA dataset as an example, the sequences of the other 10 proteins even some ribosomal sequences may also appear in it. Conversely, there are also some HA sequences in the other 10 databases. To obtain a perfect dataset of HA sequences, therefore, we select all HA sequences from all 11 datasets and delete the sequences with the same id. Then, the total number of HA sequences is 36,051, among which, the C-type HA sequences number 54, the B-type HA sequences number 3,002, the remaining 32,195 sequence are of A-type. The A-type HA sequences are classified into 16 kinds, denoted by H1, H2, until H16. In addition, some sequences are mixed and unidentified. We do not delete them from this dataset since it does not affect our statistical results. We denote C-type HA sequences by HC, B-type sequences by HB, to avoid confusion. The number of each class of HA sequences in the prepared dataset is shown as follows:

Table S2. The distribution of the numbers for 18 classes in the prepared dataset of HA sequences

| HC | HB | H1 | H2 | H3 | H4 | H5 | H6 | H7 | H8 | H9 | H10 | H11 | H12 | H13 | H14 | H15 | H16 |
| --- | --- | --- | --- | --- | --- | --- | --- | --- | --- | --- | --- | --- | --- | --- | --- | --- | --- |
| 54 | 3002 | 9837 | 315 | 14235 | 467 | 4301 | 739 | 989 | 56 | 1354 | 201 | 145 | 63 | 69 | 7 | 10 | 24 |

4．A brief introduction of MACOBMSA

To align the prepared dataset HA, it is necessary to find the multiple sequence alignment, which may be able to fast align the dataset of more than 10,000 sequences. Among the available software packages [BLAT](http://genome.cshlp.org/content/12/4/656.full) [6], [AVID](http://genome.cshlp.org/content/13/1/97.short) [7], [MUSCLE](http://nar.oxfordjournals.org/cgi/content/full/32/5/1792?ijkey=48Nmt1tta0fMg&keytype=ref) [8], [COFFEE](http://www.ncbi.nlm.nih.gov/pubmed/9682054) [9], [SAGA](http://nar.oxfordjournals.org/cgi/content/abstract/24/8/1515) [10], [MAVID](http://ukpmc.ac.uk/articlerender.cgi?artid=165629) [11], [MSAID](http://madis1.iss.ac.cn/madis.files/pub-papers/2005/37.pdf) [12], [Mauve](http://www.ncbi.nlm.nih.gov/pubmed/15231754) [13] and [MAFFT [14]](http://www.ncbi.nlm.nih.gov/pubmed/12136088), the first 8 algorithms are slow. Comparably, MAFFT is very fast. However, not all of these algorithms work for datasets this large. Therefore, we have to use the novel multiple sequence alignment software MCABMSA (unpublished). Its full name is“Multiple Compressed and Anchor-Based Multiple Sequence Alignment for the sequences of virus”.

The principle of MCABMSA is not complex, it involves the following steps:

- 1. Find the referential sequence among all input sequences, and compute the frequency of all L-length peptides.
  2. Plot the curve for the frequency of the peptide and the site of the peptide for all L-length peptides and select the anchor peptide based on the plot.
  3. On each sequence, compress each anchor peptide as one “letter”.
  4. Align all compressed sequences according to the “letters”.
  5. Select the same cluster based on the rate of the number vs the length of sequence

**The specific characteristic of MCABMSA can exactly output the aligned results into different sets if the dataset is mixed by many kinds of sequences obtained from different proteins. For example, if the dataset was mixed by sequences of Env of HIV-1 and all sequences of the influenza 11 proteins, then MCABMSA may align this dataset and exactly output the sequences of the same into one set respectively. The speed of MCABMSA is significantly fast than MUSCLE and as same as MAFFT on these datasets if the sizes of them less than 8,000. However, MCABMSA may work on the datasets more than 100,000. In order to compare the SP-score of our method with** [**MUSCLE**](http://nar.oxfordjournals.org/cgi/content/full/32/5/1792?ijkey=48Nmt1tta0fMg&keytype=ref) **and MAFFT, we use the following datasets (Data source: UniprotKB, downloaded time: June 11^th^, 2010).**

| DataSet | Capacity | The sources of the sequences |
| --- | --- | --- |
| Influenza A virus | 1,286 | HA, NA, M1, M2, NP, NS, PA, PB1, PB2 |
| HIV1 | 298 | env, gag, nef, rev, tat, vpr, vpu |

Align the subset of each protein’s sequences, we compute the SP-score for each subset and then sum all SP-scores for all proteins in each datasets, and we have the SP-scores of MUSCLE, MAFFT and MCABMSA on two datasets as below:

| DataSet | MUSCLE | MAFFT | MCABMSA |
| --- | --- | --- | --- |
| Influenza A virus | 3180359 | 3331992 | 3304046 |
| HIV1 | -642282 | -968118 | 424784 |

It shows that MCABMSA is in the ascendant.

MCABMSA involves four parameters: the length of the anchor point, the number for accepted wrong letters, the maximal relaxed number, and covering rate of anchor points. To align the amino acid sequences, the defaults of these four parameters are 7, 1, 4 and 0.8, respectively. To align the mRNA sequences, the defaults of these four parameters are 21, 3, 12 and 0.8, respectively. The user may decide to change these parameters for different purposes and different objectives (amino acid sequences, or DNA sequences).

We briefly introduce the function of each parameter focusing on the amino acid sequences as follows:

- The covering rate$r\in(0,1)$from small to large can mine the information at different levels. For clarity, we should note that the covering rate$r_{k}$of the k-th sequence is similar to the quotient of the anchor number in the k-th sequence versus the number of the benchmark sequence. We require that $r_{k}\geq r$for all k to be a very given$r$, which means that each sequence output into the same set is such that frequently only one anchor point is the same as the benchmark sequence. Thus, more sequences may be output into one set and we have a better chance to search for common peptides within a larger domain. As the parameter$r$increases to 1 (i.e.,$r=0.999$), then the sequences in a set may share all anchors, but the domain is narrowed. With the same r, two sequences in the same output set are more similar than two sequences in different output sets.
- The default of the length of the anchor point for an amino acid sequence is 7. Based on theoretical inference and prior experience, we expect 7 to be the best value, 9 to be the second best. Of course, the parameter may actually be 6 or 5 if we focus on a narrow region from site M to site N with N-M<30.
- The same anchor may not require that all 7 amino acids be the same. We may relax the restriction for a few sites and use the number for accepted wrong letters to reflect this. In practice, we find that 1 is the best value, 2 is second best.
- To generate a proper output and minimize the amount of human intervention in generating the outputs, we need to supplement these regions where there are no 7-member peptides satisfying the initial requirement of the anchor point. In practice, we allow a 7-member peptide to have more amino acids that are mismatched and the corresponding anchor point to be the second-string anchor point. This is because these regions are frequently narrow, and it is hard to find the repeated 4-member peptides within these narrow regions. Statistical experience shows that we can completely fill all regions with the second-string anchor points if the maximal number of allowed mismatched amino acids in the second-string anchor points is 4. Thus, the default of the maximal relaxed number is defined as 4.

Readers may freely download the software from <http://mathbio.nankai.edu.cn/aligneddatabase>.

5. **Supplementary Tables 1-6 for r=0.3, 0.4, 0.5, 0.6, 0.7, and 0.8 respectively and the six statements translated from the groups of vectors corresponding to these tables**

For simplicity, B-type HA sequences are denoted by HB and C-type HA sequences are denoted by HC. The main idea of the method is to classify the prepared HA datasets into different output sets using MCABMSA under each given parameter r and then compute the rates of the 18 kinds of sequences in each output set versus that in the entire HA dataset. Therefore, under each r, each output set will induce an 18-dimensional vector. For each vector, we may readily calculate the rate at which each kind of sequence is located in this output set. For example, put $r=0.001$, we align the prepared HA dataset. The number of all output sets is 7, but 5 sets contain enough sequences to allow statistical analysis. Then, for each of the five output sets, we may compute the rates of HB, HC, H1-16. Thus, we arrive at five 18-dimensional vectors as below:

Table S3. The vectors induced from the five clusters

The HA_1 vector tells us that most of H4, H14, H5 and HC sequences have at least one common conserved peptide. The HA_2 vector tells us that most of H1, H2, H6, H7, H8, H9, H10, H12, H13, H15, H16 and HB sequences have at least one common conserved peptide. The HA_1 and HA_2 vectors tell us that HB and HC sequences have no common peptide. The HA_3 vector tells us that a large rate of H3 sequences have no common conserved peptide with HC and HB. The small rates in vectors HA_5 and HA_6 have not shown enough information to make a concluding statement.

To separate H1-16 far from HB and HC, we need to choose r>0.2. Therefore, we consistently choose r=0.3, 0.4, 0.5, 0.6, 0.7 and 0.8. Then we find 6 groups of vectors and we translate each group of vectors into ordinary language in Tables 1-6 of the Supplement (item 3). According to the 6 statements translated from the 6 groups of vectors corresponding to Tables 1-6, we plot 6 figures that show the progression of the evolutionary tree for 16 kinds of sequences as r increases equidistantly (see Figure 1). The tree at r=0.8 shown in Figure 1 coincides with the tree shown in Figure 2 of reference [1]. That is, we have successfully recreated the evolutionary tree of reference [1] based on the observed sequences of Influenza A viruses using a new method.

Table S4. The vectors corresponding to r=0.3

Table S5. The vectors corresponding to r=0.4

Table S6. The vectors corresponding to r=0.5

Table S7. The vectors corresponding to r=0.6

Table S8. The vectors corresponding to r=0.7

Table S9. The vectors corresponding to r=0.8

The six statements translated from the six groups of vectors using plain language are as follows:

1. When r=0.3, vectors HA-3 and HA-4 show that H3, H7, H14 and H15 tend to strongly separate from the big cluster of all 16 types of HA while H4 and H10 do not.
2. When r=0.4, vectors HA-4 and HA-5 show group 2 (H3-H4-H7-H10-H14-H15) is formed already and the subgroup H15-H10-H7 tends to leave group 2. Vectors HA-3 and HA-6 show that group 1 (H1-H2-H5-H6-H8-H9-H11-H12-H13-H16) would be broken since that H9, H11, H13 and H16 tend to leave group 1.
3. When r=0.5, vectors HA-4 and HA-5 show that group 2 still stays together although the tendency of H7-H10-H15 to be separated is more significant than r=0.4. Vectors HA-3, HA-6 and HA-7 show that H11-H13-H16 emerges with a stronger tendency to separate from group 1. H9 also has a tendency to separate from the large cluster but it does not join the small cluster H11-H13-H16.
4. When r=0.6, vectors HA-4 and HA-5 show that H7 and H15 are far from H3-H4-H14, but H10 still keeps approaching H3-H4-H14. Vectors HA-3, HA-6 and HA-7 show that H13 and H16 are far from the cluster of group 1, H9 and H11 show different tendencies to go far although they still stay in the large cluster of group 1.
5. When r=0.7, vectors HA-4 and HA-5 show that group 2 already has been separated into two subgroups H3-H4-H14 and H7-H10-H15. While vectors HA-3, HA-6, HA-7 and HA-8 show that subgroups H11-H13-H16 and H8-H9-H12 move far from the large cluster of group 1.
6. When r=0.8, all subgroups: H1-H2-H5-H6, H8-H9-H12, H11-H13-H16, H3-H4-H14 and H7-H10-H15 further break up. Typically, H6 separates from H1-H2-H5-H6, and H1-H2-H5 has a tendency to be further separated too; H8 separates from H8-H9-H12 and H9-H12 keeps a weak connection; H11 separates from H11-H13-H16 but H13-H16 still shows a tendency to split off; H4 splits from H3-H4-H14 but H3-H14 has a further tendency to split; H10 splits from H3-H10-H15 and H3-H5 keeps stronger connection.

According to these 6 statements, we get the phylogenetic groupings of H1-16 as r increases from 0.3 to 0.8 as follows:


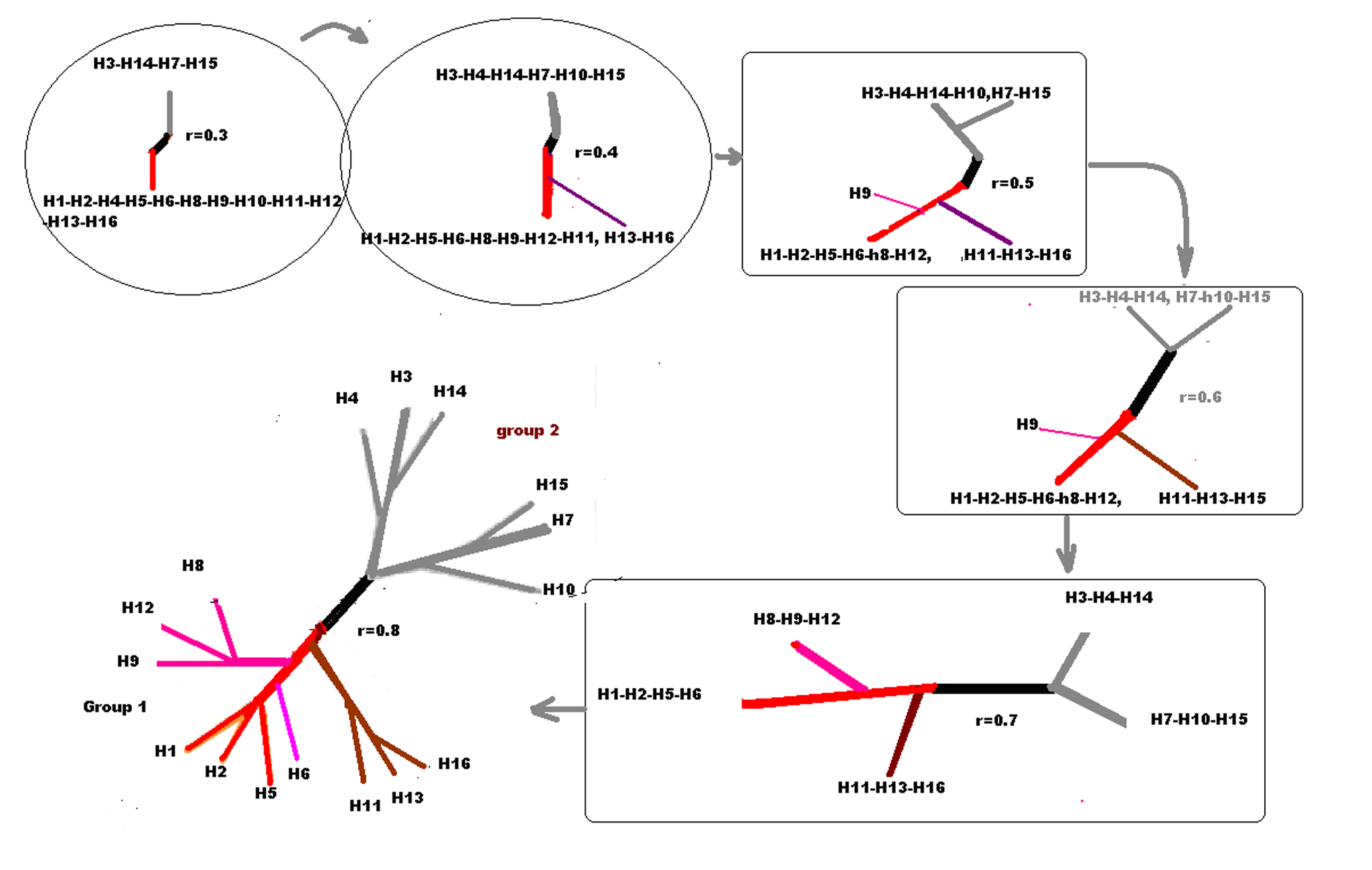


Figure S6. The phylogenetic groupings of H1-16 as *r* increases from 0.3 to 0.8

At r=0.8, the phylogenetic grouping of H1-16 is coincided to the phylogenetic grouping of H1-16 used in all references based on 39 sequences. It means we can reconstruct the phylogenetic grouping of H1-16 using a new method.

**6. Determination of the head and stem of spike using the 3D models of H1, H3 and H5**

To analyze the binding sites of the antibody shown in Figures 1and 2 of reference [4], we need to understand what fragments on the primary sequence of HA may form the stem of a spike. However, this simple question has no simple answer that can be found in the literature. Thus, we choose 1rd8, 1mql, and 2ibx from the PDB database as the 3D models of H1, H3 and H5, respectively. Then we give an intuitive definition of a stem. In the following three figures, we defined the yellow part as the stem and the white part as the head (Figures S7 (A)-(C)).


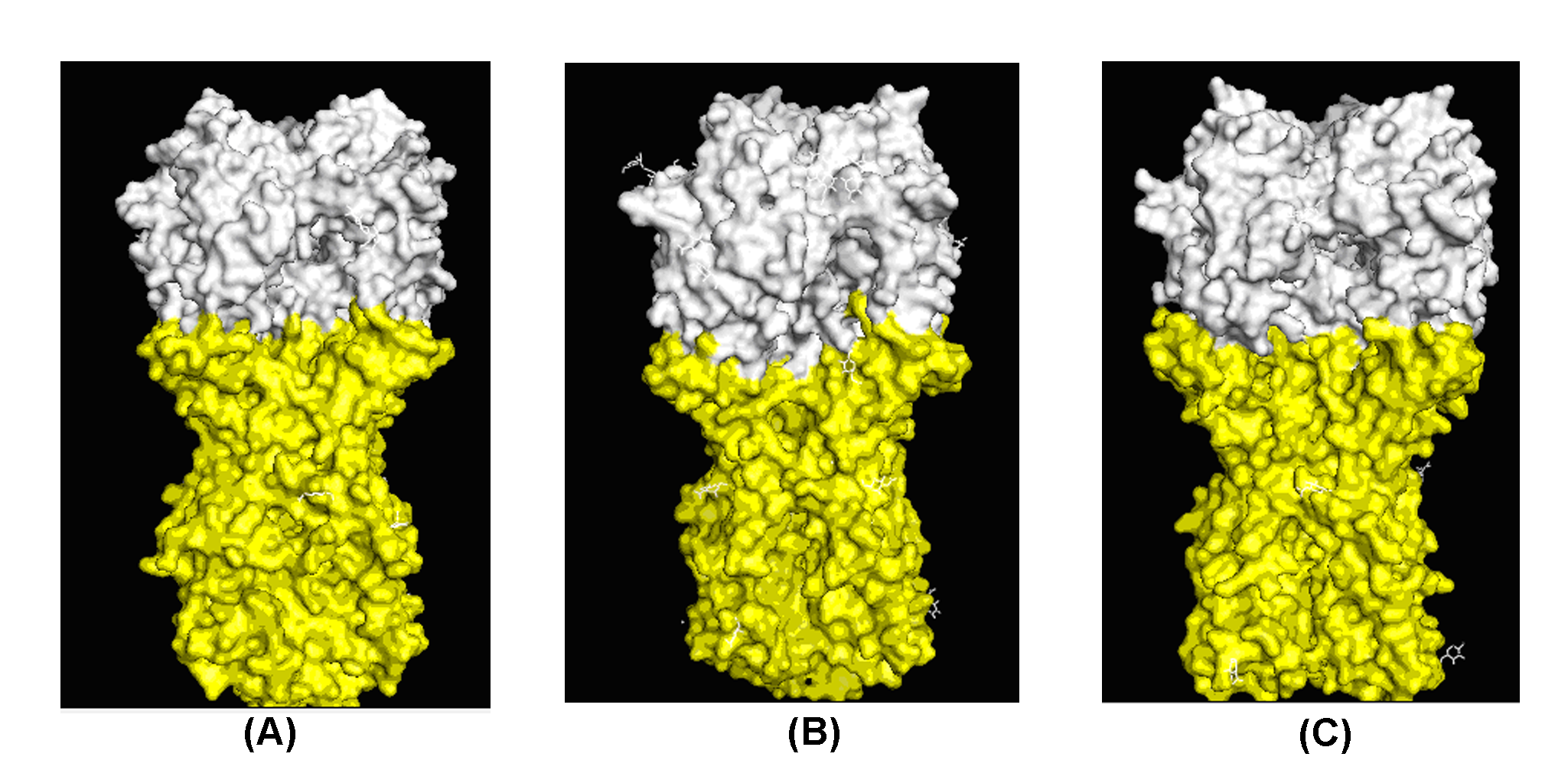


Figure S7. (A) The stem and the head of the spike for H1. (B) The stem and the head of the spike for H3. (C) The stem and the head of the spike for H5.

If we are not concerned with the angle at which we look at these figures, then the stem and the heads of the spikes are almost the same. Based on these figures, we return to the primary sequences to find the corresponding fragments. We state the obtained results below:

1. Head of spike: fragment 58-301 in the primary sequence of HA1.
2. Stem of spike: fragments 1-57 and 302-346 of HA1; fragments 1-66 and 94-196 of HA2.
3. **How to obtain the HA1 and HA2 datasets**

Using the output sets HA-2 and HA-3 at r=0.001, we find that the palindrome peptide FGAIAGF is a common peptide of A-type and B-type. Skehel and Waterfield [15] stated that FGAIAGF may be used as a marker to divide the HA1 and HA2 sets. We can also use it as a marker to generate the HA1 and HA2 datasets. However, we may only obtain 22,754 sequences if we directly use FGAIAGF as the feature to draw the sequences from the prepared HA dataset. About 10,000 HA1 sequences would be lost. Therefore, we use the following procedure to create the HA1 and HA2 datasets:

Use MCABMSA with r=0.001 to align the prepared HA dataset, and in all output sets, checking the site of the marker FGAIAGF.

Preceding FGAIAGF, there are always two hydrophobic amino acids; a common form is GLFGAIAGF. Along the vertical direction we separate the front fragment (before GLFGAIAGF, or other forms) far from the back fragment (including GLFGAIAGF and all residues after GLFGAIAGF, or other forms). We collect all of these front fragments which have more than ten amino acids into HA1, and all of these back fragments which have more than 10 amino acids into HA2. In this way, we obtain the well-defined datasets HA1 and HA2. HA1 has 31,209 sequences, and HA2 has 21,960 sequences.

Note that the number of sequences in HA2 is much smaller than the number of sequences in HA1. This is because HA2 is much more conserved than HA1, and many samples in the UniProt database only provide HA1. Thus, it is reasonable not to care about losing sequences in HA2. To show that the HA1 dataset is good enough, we compare the distribution in each of the 16 kinds in HA1 with the same kind in the prepared HA dataset given below:

Table S10. The numbers of H1-H16 appear in HA1 and the rates related to the numbers of the corresponding sequences appear in the prepared HA dataset

| H1 | H2 | H3 | H4 | H5 | H6 | H7 | H8 | H9 | H10 | H11 | H12 | H13 | H14 | H15 | H16 |
| --- | --- | --- | --- | --- | --- | --- | --- | --- | --- | --- | --- | --- | --- | --- | --- |
| 9614 | 311 | 13000 | 450 | 4279 | 731 | 975 | 47 | 1340 | 198 | 137 | 56 | 64 | 7 | 10 | 22 |
| 98% | 99% | 91% | 96% | 99% | 99% | 99% | 84% | 99% | 99% | 94% | 89% | 93% | 100% | 100% | 92% |

We see that large datasets lose some sample size, and the small ones have no sample loss. It suggests that the alternative way to rebuild HA1 and HA2 is correct.

Since the lengths of the sequences in the well-defined HA1 dataset are quite different, it means that we cannot directly read the fragments 1-57 and 301-346 from the HA1 dataset. Again, we use the conserved peptide as a significant marker of HA1 sequences to draw these fragments. Therefore, we use MCABMSA with r=0.001 again to align the well-defined dataset HA1, and then find the highly conserved peptide WGIHHP (non-frequently including WGVHHP, WAIHHP, WGINHP, WGIHHS, WGIHHQ, WGVHNP and WGVHHS). Let HA1-WGIHHP be all of these sequences having the general form WGIHHP. Then HA1-WGIHHP has 30,687 sequences distributed in each of the 16 kinds. The following table shows that the sample loss is very small if we use HA1-WGIHHP to replace HA1.

Table S11. The numbers of H1-H16 in HA1-WGIHHP and the rates relative to that in the well-defined dataset HA1

| H1 | H2 | H3 | H4 | H5 | H6 | H7 | H8 | H9 | H10 | H11 | H12 | H13 | H14 | H15 | H16 |
| --- | --- | --- | --- | --- | --- | --- | --- | --- | --- | --- | --- | --- | --- | --- | --- |
| 9529 | 268 | 12858 | 447 | 4020 | 705 | 956 | 46 | 1202 | 170 | 132 | 56 | 60 | 6 | 9 | 22 |
| 99% | 86% | 99% | 99% | 93% | 96% | 98% | 98% | 90% | 86% | 96% | 100% | 94% | 86% | 90% | 100% |

To simplify notation, we still say that HA1-WGIHHP is the HA1 dataset. Then the average length of sequences in the HA1 is about 346 (for example, USA: Memphis/10/1996 H1N1). Using WGIHHP as a marker, we further divide each HA1 sequence into two fragments: before WGIHHP and after WGIHHP. We denote them by HA1A and HA1B, respectively. We should keep in mind again that we only consider the first 58 residues (1-57) of HA1A and the last 47 residues (301-346) of HA1B.

In the same way, we use MCABMSA with r=0.001 to align the HA2 dataset. The most conserved peptide YNAELLV, located in the region 94-99 of HA2, is found. Let HA2-YNAELLV denote all of these HA2 sequences having YNAELLV (including its slightly mutated form). Then this set has 17,609 sequences. The distribution in each of the 16 kinds and the rates of the sequences located in HA2-YNAELLV relative to the sequences in HA2 are listed in Table S12:

Table S12. The distributions of the numbers the H1-H16 sequences appear in HA2-YNAELLV and HA2, and their rates

Table S12 shows that we can use YNAELLV (and its mutated forms) as a marker to divide each sequence in HA2 into two fragments: before the YNAELLV (or its mutated forms) part and the after the YNAELLV (or its mutated forms) part. We denote them by HA2A and HA2B, respectively.

That is, using the conserved peptides as markers, we draw out HA1A, HA1B, HA2A HA2B from datasets and these four sedates corresponding to these four fragments that form the stem of a spike.

**8. The site-by-site analysis of the conservation for four fragments**

We first watch the amino acids within the epitope indicated by Sui et al. It may found on page 269 of reference [2]. We call it as the five-group fashion and renamed it as table S13.

Table S13. The conservation analysis for the epitope.

The guideline of this table was stated by Sui et al as follows: Circles below residue numbers indicate estimated contribution to the binding energy at each position: red - strong; yellow - intermediate; blue - neutral. Residues without a circle are not directly involved in the epitope but are discussed in the text. Colored highlighting on the sequences indicates conservation within clusters and groups, while orange indicates high conservation or invariance. Other colors (i.e., yellow, cyan and pink) highlight residues that are cluster or subtype specific. The networks of inter-helical contacts that stabilize the fusogenic structure are indicated below the HA2 sequences. Subtypes that can be recognized/neutralized by F10 are indicated with ‘+’ on the far right. (+) or (-) indicates a predicted positive or negative binding, respectively.

We classify each of the HA1A, HA1B, HA2A and HA2B datasets into 16 sub-datasets. For example, all H1 fragments in HA1A are divided into HA1A_H1, all H2 fragments in HA1A are divided into HA1A_H2, etc. We use MCABMSA under r=0.8 to align each of 16 sub-datasets. Then, almost all fragments of a sub-dataset are well-aligned and output into one set. We process each set to get one consensus sequence based on the following rules:

- On each site, if the composition of some amino acid is larger than 0.99, then we think that all sequences on this given site share the animal acid.
- If there is no amino acid on this given site so that the composition is larger than 0.99, but the sum of largest two (or more) compositions is larger than 0.99, then we think that all sequences on the given site share these tow or more amino acids, and we show them on the same site according to their compositions from large to small.

After this processing, we have 16“new fragments”for each of HA1A, HA1B, HA2A and HA2B datasets. Then we have the site-by-site analysis of the conservation of four fragments as Table S14 and Table S15.

Table S14. The site-by-site analysis of HA1A and HA1B2

|  | 14 | 15 | 16 | 17 | 18 | 19 | 20 | 21 | 22 | 23 | 24 | 25 | 26 | 27 | 28 | 29 | 30 | 31 | 32 | 33 |
| --- | --- | --- | --- | --- | --- | --- | --- | --- | --- | --- | --- | --- | --- | --- | --- | --- | --- | --- | --- | --- |
| H2 | C | I | G | Y | H | A | N | N | S | T | E | K | V | D | T | I | L | E | R | N |
| H5 | C | I | G | Y | H | A | N | N | S | T | E | Q | V | D | T | I | M | E | K | N |
| H1 | C | I | G | Y | H | A | N | N | S | T | D | T | V | D | T | V | L | E | K | N |
| H6 | C | I | G | Y | H | A | N | N | S | T | T | Q | V | D | T | I | L | E | K | N |
|  |  |  |  |  |  |  |  |  |  |  |  |  |  |  |  |  |  |  |  |  |
| H13 | C | V | G | Y | L | S | T | N | S | S | E | K/R | V | D | T | L | L | E | N | G/D/N |
| H16 | C | I | G | Y | L | S | N | N | S | S | D | T/K | V | D | T | L | T | E | N | G |
| H11 | C | I | G | Y | L | S | N | N | S | T | E/D | K | V | D | T | I | I | E | S | N |
|  |  |  |  |  |  |  |  |  |  |  |  |  |  |  |  |  |  |  |  |  |
| H8 | C | I | G | Y | Q | S | N | N | S | T | D | T | V | N | T | L | I | E | Q | N |
| H12 | C | I | G | Y | Q | T | N | N | S | T | E | T | V | N | T | L | I | E | Q | N |
| H9 | C | I | G | Y | Q | S | T | N | S | T | E | T | V | D | T | L | T | E | N/T | N |
|  |  |  |  |  |  |  |  |  |  |  |  |  |  |  |  |  |  |  |  |  |
| H4 | C | L/M | G | H | H | A | V | P/S/A | N | G | T | M | V | K | T | L | T | D | D | Q |
| H14 | C | L | G | H | H | A | V | E | N | G | T | S | V | K | T | L | T | D | N | H |
| H3 | C | L | G | H | H | A | V | P/A | N | G | T | L/I | V | K | T | I | T | N/D | D | Q |
|  |  |  |  |  |  |  |  |  |  |  |  |  |  |  |  |  |  |  |  |  |
| H15 | C | L | G | H | H | A | V | A | N | G | T | K | V | N | T | L | T | E | R | G |
| H7 | C | L | G | H | H | A | V | A/S | N | G | T | K | V | N | T | L | T | E | R/K | G |
| H10 | C | L | G | H | H | A | V | P/S/A | N | G | T | I | V | K | T | L | T | N | E | K |

|  | 34 | 35 | 36 | 37 | 38 | 39 | 40 | 41 | 42 | 43 | 44 | 45 | 46 | 47 | 48 | 49 | 50 | 51 | 52 |
| --- | --- | --- | --- | --- | --- | --- | --- | --- | --- | --- | --- | --- | --- | --- | --- | --- | --- | --- | --- |
| H2 | V | T | V | T | H | A | K | D | I | L | E | K | T | H | N | G | K | L | C |
| H5 | V | T | V | T | H | A | Q | D | I | L | E | K | T | H | N | G | K | L | C |
| H1 | V | T | V | T | H | S | V | N | L | L | E | D | K/S | H | N | G | K | L | C |
| H6 | V | T | V | T | H | S | V | E | L | L | E | N | Q | K | E | E | R | F | C |
|  |  |  |  |  |  |  |  |  |  |  |  |  |  |  |  |  |  |  |  |
| H13 | V | P | V | T | S | S | V/I | D | L | V | E | T | N | H | T | G | T | Y | C |
| H16 | V | P | V | T | S | S | V/I | D | L | V | E | T | N | H | T | G | T | Y | C |
| H11 | V | T | V | T | S | S | V | E | L | V | E | N/T | E | H | T | G | S | F | C |
|  |  |  |  |  |  |  |  |  |  |  |  |  |  |  |  |  |  |  |  |
| H8 | V | P | V | T | Q | T | M | E | L | V | E | T | E | K | H | P | A | Y | C |
| H12 | V | P | V | T | Q | V | E | E | L | V | H | G | G | I | D | P | I | L | C |
| H9 | V | P | V | T | H | A | K | E | L | L | H | T | E | H | N | G | M | L | C |
|  |  |  |  |  |  |  |  |  |  |  |  |  |  |  |  |  |  |  |  |
| H4 | I/V | E | V | V | T/A | A | Q | E | L | V | E | S | Q | H/N | L | P | E | L | C |
| H14 | V | E | V | V | S | A | K | E | L | V | E | T | N | H | T | D | E | L | C |
| H3 | I | E | V | T | N | A | T | E | L | V | Q | S | S/I | S | T/I | G | K/G/R/E | I | C |
|  |  |  |  |  |  |  |  |  |  |  |  |  |  |  |  |  |  |  |  |
| H15 | V | E | V | V | N | A | T | E | T | V | E | I | T | G | I | D | K | V | C |
| H7 | I/V | E | V | V | N | A | T | E | T | V | E | T/R | A/T | N | I/V | K/P | K/R | I | C |
| H10 | E | E | V | T | N | A | T | E | T | V | E | S | K | S | L | D | R | L | C |

|  | 277 | 278 | 279 | 280 | 281 | 282 | 283 | 284 | 285 | 286 |
| --- | --- | --- | --- | --- | --- | --- | --- | --- | --- | --- |
| H2 | C | E | T | K | C | Q | T | P | L | G |
| H5 | C | ND | T | K | C | Q | T | P | IM | G |
| H1 | C | ND | TA | TK | C | Q | T | P | KQ | G |
| H6 | C | D | A | TV | C | Q | T | I | A | G |
|  |  |  |  |  |  |  |  |  |  |  |
| H13 | C | N | T | K | C | Q | T | S | V | G |
| H16 | C | N | T | K | C | Q | T | S | MVL | G |
| H11 | C | S | T | K | C | Q | ST | E | I | G |
|  |  |  |  |  |  |  |  |  |  |  |
| H8 | C | HN | T | K | C | Q | T | Y | A | G |
| H12 | C | VT | T | E | C | Q | L | N | E | G |
| H9 | C | VT | V | Q | C | Q | T | E | KR | G |
|  |  |  |  |  |  |  |  |  |  |  |
| H4 | C | V | S | K | C | H | T | D | RK | G |
| H14 | C | T | S | P | C | L | T | D | K | G |
| H3 | C | NV | S | E | C | I | T | P | N | G |
|  |  |  |  |  |  |  |  |  |  |  |
| H15 | C | E | G | E | C | F | Y | S | G | G |
| H7 | C | EG | G | D | C | FY | H | S | G | G |
| H10 | C | E | S | K | C | F | W | KR | G | G |

|  | 287 | 288 | 289 | 290 | 291 | 292 | 293 | 294 | 295 | 296 | 297 | 298 | 299 | 300 | 301 | 302 | 303 | 304 | 305 | 306 | 307 | 308 | 309 |
| --- | --- | --- | --- | --- | --- | --- | --- | --- | --- | --- | --- | --- | --- | --- | --- | --- | --- | --- | --- | --- | --- | --- | --- |
| H2 | A | I | N | T | T | L | P | F | H | N | I/V | H | P | L | T | I | G | E | C | P | K | Y | V |
| H5 | A | I | N | S | S | M | P | F | H | N | I | H | P | L | T | I | G | E | C | P | K | Y | V |
| H1 | A | I | N | T/S | S | L | P | F | Q | N | I/V | H | P | I/V | T | I | G | K/E | C | P | K | Y | V |
| H6 | V | L/I | R | T | N | K | T | F | Q | N | V | S | P | L | W | I | G | E | C | P | K | Y | V |
|  |  |  |  |  |  |  |  |  |  |  |  |  |  |  |  |  |  |  |  |  |  |  |  |
| H13 | G | I | N | T | N | K/R | T | F | Q | N | I | E/D | R/K | N | A | L | G | D/N | C | P | K | Y | I |
| H16 | G | I/V | N | T | N | K | T | F | Q | N | I | E/D | R | N | A | L/I | G | D | C | P | K | Y | I |
| H11 | G | I | N | T | N | R/K | S | F | H | N/S | V | H | R | N | T | I | G | D | C | P | K | Y | V |
|  |  |  |  |  |  |  |  |  |  |  |  |  |  |  |  |  |  |  |  |  |  |  |  |
| H8 | A | I | N | S | S | K | P | F | Q | N | A | S | R | H | Y | M | G | E | C | P | K | Y | V |
| H12 | V | M | N | T | S | K | P | F | Q | N | T | S | K | H | Y | I | G | K | C | P | K | Y | I |
| H9 | G | L | N | T/S | T | L | P | F | H/Q | N | V/I | S | K | Y | A | F | G | N | C | P | K | Y | V/I |
|  |  |  |  |  |  |  |  |  |  |  |  |  |  |  |  |  |  |  |  |  |  |  |  |
| H4 | S | I/L | S/T | T | T | K | P | F | Q | N | I | S | R | I | S/A | I | G | D | C | P | K | Y | V |
| H14 | S | I | Q | S | D | K | P | F | Q | N | V | S | R | I | A | I | G | N | C | P | K | Y | V |
| H3 | S | I | P | N | D | K | P | F | Q | N | V | N | R | I | T | Y | G | A | C | P | R | Y | V |
|  |  |  |  |  |  |  |  |  |  |  |  |  |  |  |  |  |  |  |  |  |  |  |  |
| H15 | T | I | N | S | P | L | P | F | Q | N | I | D | S | R | A | V | G | K | C | P | R | Y | V |
| H7 | T | I | V/I | S | S/N | L | P | F | Q | N | I | N | S/P | R | T/A | V | G | K | C | P | R | Y | V |
| H10 | S | I | N | T | K | L | P | F | Q | N | L | S | P | R | T | V | G | Q | C | P | K | Y | V |

|  | 310 | 311 | 312 | 313 | 314 | 315 | 316 | 317 | 318 | 319 | 320 | 321 | 322 | 323 | 324 | 325 | 326 | 327 | 328 | 329 |
| --- | --- | --- | --- | --- | --- | --- | --- | --- | --- | --- | --- | --- | --- | --- | --- | --- | --- | --- | --- | --- |
| H2 | K | S | E/D | R/K | L | V | L | A | T | G | L | R | N | V | P | Q | I | E | S | R |
| H5 | K | S | N/D | R/K | L | V | L | A | T | G | L | R | N | S/V | P | Q | R/G | E | R/T | R |
| H1 | K/R | S | T/A | K | L | R | L/M | A/V | T | G | L | R | N | V/I | P | S | I | Q | S | R |
| H6 | K | S | E/K | S | L | R | L | A | T | G | L | R | N | V | P | Q | I | E | T | R |
|  |  |  |  |  |  |  |  |  |  |  |  |  |  |  |  |  |  |  |  |  |
| H13 | K | S | G | Q | L | K | L | A | T | G | L | R | N | V | P | A | I | S | N | R |
| H16 | K | S | G | Q | L | K | L | A | T | G | L | R | N | V | P | S | I | G/N/V | E | R |
| H11 | N | V | K | S | L | K | L | A | T | G | L | R | N | V | P | A | I | A | T/S | R |
|  |  |  |  |  |  |  |  |  |  |  |  |  |  |  |  |  |  |  |  |  |
| H8 | K | K | A/E | S | L | R | L | A | V | G | L | R | N | T | P | S | I/V | E | P | K/R |
| H12 | P | S | G | S | L | K | L | A | I | G | L | R | N | V | P | Q | A/V | Q | N/D | R |
| H9 | G | V | K | S | L | K | L | A | V | G | L | R | N | V | P | A | R | S | S | R |
|  |  |  |  |  |  |  |  |  |  |  |  |  |  |  |  |  |  |  |  |  |
| H4 | K | Q | G | S | L | K | L | A | T | G | M | R | N | I | P | E | K | A | T | R |
| H14 | K | Q | G | S | L | M | L | A | T | G | M | R | N | I | P | G | K | Q | A | K |
| H3 | K | Q | N | T | L | K | L | A | T | G | M | R | N | V | P | E | K | Q | T | R |
|  |  |  |  |  |  |  |  |  |  |  |  |  |  |  |  |  |  |  |  |  |
| H15 | K | Q | S | S | L | P | L | A | L | G | M | K | N | V | P | E | K | I |  |  |
| H7 | K | Q | K/E/T | S | L | L | L | A | T | G | M | R/K | N | V | P | E | K/N/I | P | K | P/G/T |
| H10 | N | K | K/R | S | L | L/M | L | A | T | G | M | R | N | V | P | E | V/I | V/M | Q | G |

Table S15. The site-by-site analysis of HA2A and HA2 B

|  | 3 | 4 | 5 | 6 | 7 | 8 | 9 | 10 | 11 | 12 | 13 | 14 | 15 | 16 | 17 | 18 | 19 | 20 | 21 | 22 | 23 | 24 | 25 | 26 | 27 | 28 | 29 | 30 | 31 |
| --- | --- | --- | --- | --- | --- | --- | --- | --- | --- | --- | --- | --- | --- | --- | --- | --- | --- | --- | --- | --- | --- | --- | --- | --- | --- | --- | --- | --- | --- |
| H2 | F | G | A | I | A | G | F | I | E | G | G | W | Q | G | M | V | D | G | W | Y | G | Y | H | H | S | N | D | Q | G |
| H5 | F | G | A | I | A | G | F | I | E | G | G | W | Q | G | M | V | D | G | W | Y | G | Y | H | H | S | N | E | Q | G |
| H1 | F | G | A | I | A | G | F | I | E | G | G | W | T | G | M | V | D | G | W | Y | G | Y | H | H | Q | N | E | Q | G |
| H6 | F | G | A | I | A | G | F | I | E | G | G | W | T | G | M | I | D | G | W | Y | G | Y | H | H | E | N | S | Q | G |
|  |  |  |  |  |  |  |  |  |  |  |  |  |  |  |  |  |  |  |  |  |  |  |  |  |  |  |  |  |  |
| H13 | F | G | A | I | A | G | F | I | E | G | G | W | P | G | L | I | N | G | W | Y | G | F | Q | H | Q | N | E | Q | G |
| H16 | F | G | A | I | A | G | F | I | E | G | G | W | P | G | L | I | N | G | W | Y | G | F | Q | H | Q | N | E | Q | G |
| H11 | F | G | A | I | A | G | F | I | E | G | G | W | P | G | L | I | N | G | W | Y | G | F | Q | H | R | N | E | E | G |
|  |  |  |  |  |  |  |  |  |  |  |  |  |  |  |  |  |  |  |  |  |  |  |  |  |  |  |  |  |  |
| H8 | F | G | A | I | A | G | F | I | E | G | G | W | S | G | M | I | D | G | W | Y | G | F | H | H | S | N | S | E | G |
| H12 | F | G | A | I | A | G | F | I | E | G | G | W | P | G | L | V | A | G | W | Y | G | F | Q | H | Q | N | A | E | G |
| H9 | F | G | A | I | A | G | F | I | E | G | G | W | S | G | L | V | A | G | W | Y | G | F | Q | H | S | N | D | Q | G |
|  |  |  |  |  |  |  |  |  |  |  |  |  |  |  |  |  |  |  |  |  |  |  |  |  |  |  |  |  |  |
| H4 | F | G | A | I | A | G | F | I | E | N | G | W | Q | G | L | I | D | G | W | Y | G | F | R | H | Q | N | A | E | G |
| H14 | F | G | A | I | A | G | F | I | E | N | G | W | Q | G | L | I | D | G | W | Y | G | F | R | H | Q | N | A | E | G |
| H3 | F | G | A | I | A | G | F | I | E | N | G | W | E | G | M | V/I | D | G | W | Y | G | F | R | H | Q | N | S | E | G |
|  |  |  |  |  |  |  |  |  |  |  |  |  |  |  |  |  |  |  |  |  |  |  |  |  |  |  |  |  |  |
| H15 | F | G | A | I | A | G | F | I | E | N | G | W | E | G | L | I | D | G | W | Y | G | F | R | H | Q | N | A | Q | G |
| H7 | F | G | A | I | A | G | F | I | E | N | G | W | E | G | L | I | D/N | G | W | Y | G | F | R | H | Q | N | A | Q | G |
| H10 | F | G | A | I | A | G | F | I | E | N | G | W | E | G | M | V | D | G | W | Y | G | F | R | H | Q | N | A | Q | G |

|  | 32 | 33 | 34 | 35 | 36 | 37 | 38 | 39 | 40 | 41 | 42 | 43 | 44 | 45 | 46 | 47 | 48 | 49 | 50 | 51 | 52 | 53 | 54 | 55 | 56 | 57 | 58 |
| --- | --- | --- | --- | --- | --- | --- | --- | --- | --- | --- | --- | --- | --- | --- | --- | --- | --- | --- | --- | --- | --- | --- | --- | --- | --- | --- | --- |
| H2 | S | G | Y | A | A | D | K | E | S | T | Q | K | A | I/F | D | G | I | T | N | K | V | N | S | V | I | E | K |
| H5 | S | G | Y | A | A | D | K | E | S | T | Q | K | A | I | D | G | V | T | N | K | V | N | S | I | I | D | K |
| H1 | S | G | Y | A | A | D | L/Q | K | S | T | Q | N | A | I | D/N | E/G | I | T | N | K | V | N | S | V | I | E | K |
| H6 | S | G | Y | A | A | D | R/K | E | S | T | Q | K | A | I/V | D/N | G/K | I | T | N | K | V | N | S | I | I | D | K |
|  |  |  |  |  |  |  |  |  |  |  |  |  |  |  |  |  |  |  |  |  |  |  |  |  |  |  |  |
| H13 | V | G | I/M | A | A | D | K | E | S | T | Q | K | A | I | D | Q | I | T | T | K | I | N | N | I | I | D/E | K |
| H16 | T | G | I | A | A | D | K | A/T | S | T | Q | K | A | I | N/D | E | I | T | T | K | I | N | N | I | I | E | K |
| H11 | T | G | I | A | A | D | K | E | S | T | Q | K | A | I | D | Q | I | T | S | K | V | N | N | I | V | D | R |
|  |  |  |  |  |  |  |  |  |  |  |  |  |  |  |  |  |  |  |  |  |  |  |  |  |  |  |  |
| H8 | T | G | M | A | A | D | Q | K | S | T | Q | E | A | I | D | K | I | T | N | K | V | N | N | I | V | D | K |
| H12 | T | G | I | A | A | D | R | D | S | T | Q | K | A | I | D | N | M | Q | N | K | L | N | N | V | I | D | K |
| H9 | V | G | M | A | A | D | R | D | S | T | Q | K | A | I | D | K | I | T | S | K | V | N | N | I | V | D | K |
|  |  |  |  |  |  |  |  |  |  |  |  |  |  |  |  |  |  |  |  |  |  |  |  |  |  |  |  |
| H4 | T | G | T | A | A | D | L | K | S | T | Q | A | A | I | D | Q | I | N | G | K | L | N | R | L | I | E | K |
| H14 | T | G | T | A | A | D | L | K | S | T | Q | A | A | I | D | Q | I | N | G | K | L | N | R | L | I | E | K |
| H3 | T/I/R | G | Q | A | A | D | L | K | S | T | Q | A | A | I | D/N | Q | I | N | G | K | L | N | R | L/V | I | E/G | K |
|  |  |  |  |  |  |  |  |  |  |  |  |  |  |  |  |  |  |  |  |  |  |  |  |  |  |  |  |
| H15 | Q | G | T | A | A | D | Y | K | S | T | Q | A | A | I | D | Q | I | T | G | K | L | N | R | L | I | E | K |
| H7 | E | G | T | A | A | D | Y | K | S | T | Q | S | A | I | D | Q | I | T | G | K | L | N | R | L | I | E/G/D | K |
| H10 | T | G | Q | A | A | D | Y | K | S | T | Q | A | A | I | D | Q | I | T | G | K | L | N | R | L | I | E | K |

|  | 59 | 60 | 61 | 62 | 63 | 64 | 65 | 66 | 67 | 68 | 69 | 70 | 71 | 72 | 73 | 74 | 75 | 76 | 77 | 78 | 79 | 80 | 81 | 82 | 83 | 84 | 85 | 86 | 87 |
| --- | --- | --- | --- | --- | --- | --- | --- | --- | --- | --- | --- | --- | --- | --- | --- | --- | --- | --- | --- | --- | --- | --- | --- | --- | --- | --- | --- | --- | --- |
| H2 | M | N | T | Q | F | E | A | V | G | K | E | F | N/S | N | L | E | R/K | R | L | E | N | L | N | K | K | M | E | D | G |
| H5 | M | N | T | Q | F | E | A | V | G | R | E | F | N | N | L | E | R | R | I | E | N | L | N | K | K | M | E | D | G |
| H1 | M | N | T | Q | F | T | A | V | G | K | E | F | N | H/K | L | E | K/R | R | I/M | E | N | L | N | K | K | V | D | D | G |
| H6 | M | N | T | Q | F | E | A | V | D/E | H | E | F | S | N | L | E | R | R | I | D/G | N | L | N | K | R | M | E/Q | D | G |
|  |  |  |  |  |  |  |  |  |  |  |  |  |  |  |  |  |  |  |  |  |  |  |  |  |  |  |  |  |  |
| H13 | M | N | G | N | Y | D | S | I | R | G | E | F | N/S | Q | V | E | K/Q | R | I | N | M | L | A | D | R | I | D | D | A |
| H16 | M | N | G | N | Y | D | S | I | R | G | E | F | N | Q | V | E | K | R | I | N | M | L/I | A | D | R | V | D | D | A |
| H11 | M | N | T | N | F | E | S | V | Q | H | E | F | S | E | I | E | E | R | I | N | Q | L | S | K | H | V | D | D | S |
|  |  |  |  |  |  |  |  |  |  |  |  |  |  |  |  |  |  |  |  |  |  |  |  |  |  |  |  |  |  |
| H8 | M | N | R | E | F | E | V | V | N | H | E | F | S | E | V | E | K | R | I | N | M | I | N | D | K | I | D | D | Q |
| H12 | M | N | K | Q | F | E | V | V | N | H | E | F | S | E | V | E | S | R | I | N | M | I | N | S | K | I | D | D | Q |
| H9 | M | N | K | Q | Y | E | I | I | D | H | E | F | S | E | V/I | E | T/A | R | L | N | M | I | N | N | K | I | D | D | Q |
|  |  |  |  |  |  |  |  |  |  |  |  |  |  |  |  |  |  |  |  |  |  |  |  |  |  |  |  |  |  |
| H4 | T | N | E | K | Y | H | Q | I | E | K | E | F | E | Q | V | E | G | R | I | Q | D | L | E | K | Y | V | E | D | T |
| H14 | T | N | E | K | Y | H | Q | I | E | K | E | F | E | Q | V | E | G | R | I | Q | D | L | E | K | Y | V | E | D | T |
| H3 | T | N | E | K | F | H | Q | I | E | K | E | F | S | E | V | E | G | R | I | Q | D | L | E | K | Y | V | E | D | T |
|  |  |  |  |  |  |  |  |  |  |  |  |  |  |  |  |  |  |  |  |  |  |  |  |  |  |  |  |  |  |
| H15 | T | N | K | Q | F | E | L | I | D | N | E | F | T | E | V | E | Q | Q | I | G | N | V | I | N | W | T | R | D | S |
| H7 | T | N | Q | Q | F | E | L | I | D | N | E | F | N/T | E | I/V | E | Q/K | Q | I | G | N | V | I | N | W | T | R | D | S/A |
| H10 | T | N | T | E | F | E | S | I | E | S | E | F | S | E | I | E | H | Q | I | G | N | V | I | N | W | T | K | D | S |

|  | 88 | 89 | 90 | 91 | 92 | 93 | 94 | 95 | 96 | 97 | 98 | 99 | 100 | 101 | 102 | 103 | 104 | 105 | 106 | 107 | 108 | 109 | 110 | 111 | 112 | 113 |
| --- | --- | --- | --- | --- | --- | --- | --- | --- | --- | --- | --- | --- | --- | --- | --- | --- | --- | --- | --- | --- | --- | --- | --- | --- | --- | --- |
| H2 | Y | N | A | E | L | L | V | L | M | E | N | E | R | T | L | D | F | H | D | S | N | V | K | N | L | Y |
| H5 | Y | N | A | E | L | L | V | L | M | E | N | E | R | T | L | D | F | H | D | S | N | V | K | N | L | Y |
| H1 | Y | N | A | E | L | L | V | L | L | E | N | E | R | T | L | D | Y/F | H | D | S | N | V | K | N | L | Y |
| H6 | Y | N | A | E | L | L | V | L | L | E | N | E | R | T | L | D | L/M | H | D | A | N | V | K | N | L | Y/H |
|  |  |  |  |  |  |  |  |  |  |  |  |  |  |  |  |  |  |  |  |  |  |  |  |  |  |  |
| H13 | Y | N | A | K | L | L | V | L | L | E | N | D | K | T | L | D | M | H | D | A | N | V | R | N | L | H |
| H16 | Y | N | A | K | L | L | V | L | I/L | E | N | D/G | R | T | L | D | L | H | D | A | N | V | R/K | N | L | H |
| H11 | Y | N | A | Q | L | L | V | L | L | E | N | E | K | T | L | D | L | H | D | S | N | V | R | N | L | H |
|  |  |  |  |  |  |  |  |  |  |  |  |  |  |  |  |  |  |  |  |  |  |  |  |  |  |  |
| H8 | Y | N | A | E | L | L | V | L | L | E | N | Q | K | T | L | D | E | H | D | S | N | V | K | N | L | F |
| H12 | Y | N | A | E | L | L | V | L | L | E | N | Q | K | T | L | D | E | H | D | A | N | V | R | N | L | H |
| H9 | Y | N | A | E | L | L | V | L | L | E | N | Q | K | T | L | D | E | H | D | A | N | V | N | N | L | Y |
|  |  |  |  |  |  |  |  |  |  |  |  |  |  |  |  |  |  |  |  |  |  |  |  |  |  |  |
| H4 | Y | N | A | E | L | L | V | A | L | E | N | Q | H | T | I | D | V | T | D | S | E | M | N | K | L | F |
| H14 | Y | N | A | E | L | L | V | A | L | E | N | Q | H | T | I | D | V | T | D | S | E | M | N | K | L | F |
| H3 | Y | N | A | E | L | L | V | A | L | E | N | Q | H | T | I | D | L | T | D | S | E | M | N | K | L | F |
|  |  |  |  |  |  |  |  |  |  |  |  |  |  |  |  |  |  |  |  |  |  |  |  |  |  |  |
| H15 | Y | N | A | E | L | L | V | A | M | E | N | Q | H | T | I | D | L | A | D | S | E | M | N | K | L | Y |
| H7 | Y | N | A | E | L | L | V | A | M | E | N | Q | H | T | I | D | L | A | D | S | E | M | N/S | K | L | Y |
| H10 | Y | Q | A | E | L | L | V | A | M | E | N | Q | H | T | I | D | M | A | D | S | E | M | L | N | L | Y |

|  | 121 | 122 | 123 | 124 | 125 | 126 | 127 | 128 | 129 | 130 | 131 | 132 | 133 | 134 | 135 | 136 | 137 | 138 | 139 | 140 | 141 | 142 | 143 | 144 | 145 |
| --- | --- | --- | --- | --- | --- | --- | --- | --- | --- | --- | --- | --- | --- | --- | --- | --- | --- | --- | --- | --- | --- | --- | --- | --- | --- |
| H2 | K | V | R | M | Q | L | R | D | N | A/V | K | E | L | G | N | G | C | F | E | F | Y | H | K | C | D |
| H5 | K | V | R | L | Q | L | R | D | N | A | K | E | L | G | N | G | C | F | E | F | Y | H | K/R | C | D |
| H1 | K | V | R/K | S | Q | L | K | N | N | A | K | E | I | G | N | G | C | F | E | F | Y | H | K | C | D/N |
| H6 | K | V | K | S | Q | L | R | D | N | A | N/K | D | L | G | N | G | C | F | E | F | W | H | K | C | D |
|  |  |  |  |  |  |  |  |  |  |  |  |  |  |  |  |  |  |  |  |  |  |  |  |  |  |
| H13 | Q | V | R | R | E/A/T/V | L | K | T/D | N | A | I/V | D | E | G | N | G | C | F | E | L | L | H | K | C | N |
| H16 | Q | V/I | K | R | A | L | K | N/D/S | N | A | I | D | E | G | D | G | C | F | N/S | L | L | H | K | C | N |
| H11 | K | V | R | R | M | L | K | D | N | A | K | D | E | G | N | G | C | F | T | F | Y | H | K | C | D |
|  |  |  |  |  |  |  |  |  |  |  |  |  |  |  |  |  |  |  |  |  |  |  |  |  |  |
| H8 | E | V | K/R | R | R | L | S | A/T | N | A | I | D | A/T | G | N | G | C | F | D | I | L | H | K | C | N/D |
| H12 | R | V/I | R | R | V | L | R | E | N | A | I | D | T | G | D | G | C | F | E | I | L | H | K/R | C | D |
| H9 | K | V | K | R | A | L | G | S | N | A | V/M | E | D | G | K | G | C | F | E | L | Y | H | K | C | D |
|  |  |  |  |  |  |  |  |  |  |  |  |  |  |  |  |  |  |  |  |  |  |  |  |  |  |
| H4 | R | V | R | R | Q | L | R | E | N | A | E | D | K | G | N | G | C | F | E | I | F | H | Q/K | C | D |
| H14 | R | V | R | R | Q | L | R | E | N | A | E | D | Q | G | N | G | C | F | E | I | F | H | Q | C | D |
| H3 | R/K | T | K/R | K/R | Q | L | R | E | N | A | E | D | M | G | N | G | C | F | K | I | Y | H | K | C | D |
|  |  |  |  |  |  |  |  |  |  |  |  |  |  |  |  |  |  |  |  |  |  |  |  |  |  |
| H15 | R | V | R | R | Q | L | R | E | N | A | E | E | D | G | T | G | C | F | E | I | F | H | R | C | D |
| H7 | R | V | R/K | K/R | Q | L | R | E | N | A | E | E | D | G | T | G | C | F | E | I | F | H | K | C | D |
| H10 | R | V | R | K | Q | L | R | Q | N | A | E | E | D | G | K | G | C | F | E | I | Y | H | K/A | C | D |

|  | 146 | 147 | 148 | 149 | 150 | 151 | 152 | 153 | 154 | 155 | 156 | 157 | 158 | 159 | 160 | 161 | 162 | 163 | 164 | 165 | 166 | 167 | 168 | 169 | 170 |
| --- | --- | --- | --- | --- | --- | --- | --- | --- | --- | --- | --- | --- | --- | --- | --- | --- | --- | --- | --- | --- | --- | --- | --- | --- | --- |
| H2 | D | E | C | M | N | S | V | K/R | N | G | T | Y | D | Y | P | K | Y | E | E | E | S | K | L | N | R |
| H5 | N | E | C | M | E | S | V | R | N | G | T | Y | D | Y | P | Q | Y | S | E | E | A | R | L | K/N | R |
| H1 | N/D | T/E | C | M | E | S | V | K | N | G | T | Y | D | Y | P | K | Y | S | E | E | A/S | K | L | N | R |
| H6 | N | E | C | I | E | S | V | K | N | G | T | Y | D/N | Y | P | K | Y | Q | D/T/E/G/A | E | S | K/R | L | N | R |
|  |  |  |  |  |  |  |  |  |  |  |  |  |  |  |  |  |  |  |  |  |  |  |  |  |  |
| H13 | D | S | C | M | E | T | I | R | N | G | T | Y | N/D | H | T | E | Y | A/E | E | E | S | K | L | K | R |
| H16 | D | S | C | M | E | T | I | R | N | G | T | Y | N | H | E | D | Y | K/R | E | E | S | Q | L | K | R |
| H11 | N | E | C | I | E | K/R | V | R | N | G | T | Y | D | H | K | E | F | E | E | E | S | K/R | L/I | N | R |
|  |  |  |  |  |  |  |  |  |  |  |  |  |  |  |  |  |  |  |  |  |  |  |  |  |  |
| H8 | N | E | C | M | E | T | I | K | N | G | T | Y | N/D | H | K | E | Y | E | E | E | A | K | L | E | R |
| H12 | N/D | N | C | M | D | T | I | R | N | G | T | Y | N | H | K/Q/R | E | Y | E | E | E | S | K | I | E | R |
| H9 | D | Q | C | M | E | T | I | R | N | G | T | Y | N | R | R | K | Y | K/Q | E | E | S | R/K | L | E | R |
|  |  |  |  |  |  |  |  |  |  |  |  |  |  |  |  |  |  |  |  |  |  |  |  |  |  |
| H4 | N | N | C | I | E | S | I | R | N | G | T | Y | D | H | D | I | Y | R | D | E | A | I | N | N | R |
| H14 | N | N | C | I | E | S | I | R | N | G | T | Y | D | H | N | I | Y | R | D | E | A | I | N | N | R |
| H3 | N | A | C | I | G/E | S | I | R | N | G | T | Y | D | H | D | V | Y | R | D | E | A | L | N | N | R |
|  |  |  |  |  |  |  |  |  |  |  |  |  |  |  |  |  |  |  |  |  |  |  |  |  |  |
| H15 | D | Q | C | M | E | S | I | R | N | N | T | Y | N | H | T | E | Y | R | Q | E | A | L | Q | N | R |
| H7 | D | Q/D | C | M | E/A | S | I | R | N | N | T | Y | D | H | T/S | Q/K | Y | R | T/E | E | S/A | L/M | Q | N | R |
| H10 | D | N/S | C | M | E | S | I | R | N | N | T | Y | D | H | T/S | Q | Y | R | E | E | A | L | L | N | R |

|  | 171 | 172 | 173 | 174 | 175 | 176 | 177 | 178 | 179 | 180 | 181 | 182 | 183 | 184 | 185 | 186 | 187 | 188 | 189 | 190 | 191 | 192 | 193 | 194 | 195 | 196 |
| --- | --- | --- | --- | --- | --- | --- | --- | --- | --- | --- | --- | --- | --- | --- | --- | --- | --- | --- | --- | --- | --- | --- | --- | --- | --- | --- |
| H2 | N | E | I | K | G | V | K | L | S | N/S | M | G | V | Y | Q | I | L | A | I | Y | A | T | V | A | G | S |
| H5 | E | E | I | S | G | V | K | L | E | S | I/M | G | T/I | Y | Q | I | L | S | I | Y | S | T | V | A | S | S |
| H1 | E | E/K | I | D | G | V | K | L | E | S | T/M | R/G | I/V | Y | Q | I | L | A | I | Y | S | T | V | A | S | S |
| H6 | Q/L | E/K/T | I | E | S | V | K | L | E | N | L | G | V | Y | Q | I | L | A | I | Y | S | T | V | S | S | S |
|  |  |  |  |  |  |  |  |  |  |  |  |  |  |  |  |  |  |  |  |  |  |  |  |  |  |  |
| H13 | Q | E | I | E/N | G | I | K | L | K | S | E/D | D | N/S | V | Y | K | A | L | S | I | Y | S | C | I | A | S |
| H16 | Q | E | I | E | G | I | K | L | K | T | E | D | N | V | Y | K | I/V | L | S | I | Y | S | C | I | A | S |
| H11 | Q | E | I | E | G | V | K | L | D | S | N/S | G | N | V | Y | K | I | L | S | I | Y | S | C | I | A | S |
|  |  |  |  |  |  |  |  |  |  |  |  |  |  |  |  |  |  |  |  |  |  |  |  |  |  |  |
| H8 | S | K | I | N | G | V | K | L | E | E | N | T | T | Y | K | I | L | S | I | Y | S | T | V | A | A | S |
| H12 | Q | K | I | N | G | V | K | L | E | E | N | S | T | Y | K | I | L | S | I | Y | S | S | V | A | S | S |
| H9 | Q | K | I | E | G | V | K | L | E | S | E | G | T | Y | K | I | L | T | I | Y | S | T | V | A | S | S |
|  |  |  |  |  |  |  |  |  |  |  |  |  |  |  |  |  |  |  |  |  |  |  |  |  |  |  |
| H4 | F | Q | I | Q | G | V | K | L | T | Q | G | Y | K | D | I | I | L | W | I | S | F | S | I | S | C | F |
| H14 | I | K | I | N | P | V | T | L | T | M | G | Y | K | D | I | I | L | W | I | S | F | S | M | S | C | F |
| H3 | F | Q | I | K | G | V | E | L | K | S | G | Y | K | D | W | I | L | W | I | S | F | A | I | S | C | F |
|  |  |  |  |  |  |  |  |  |  |  |  |  |  |  |  |  |  |  |  |  |  |  |  |  |  |  |
| H15 | I | M | I | N | P | V | K | L | S | S | G | Y | K | D | V | I | L | W | F | S | F | G | A | S | C | V |
| H7 | I | Q | I | D | P | V | K | L | S | S | G | Y | K | D | I/V | I | L | W | F | S | F | G | A | S | C | F |
| H10 | L | N | I | N | P/S | V | K | L | S | S | G | Y | K | D | V/I | I | L | W | F | S | F | G | A | S | C | F |

For the sake of convenience, we should make some clarifications for the supplement tables S14 and S15 as follows:

First, we also highlight with green a site if it is unchanged for all 16 kinds; with yellow if a site is unchanged for more than one of the five subgroups but not for all 16 kinds; with red if a site is just unchanged for a subgroup. Otherwise, we keep it in the original form.

Second, we note differences involving the sites between tables S14 and S15. This is due to that startup residue we use as the common M for most sequences.

Incidentally, the dipeptide NL (at the cross of 291-292 and H1, Table S11) should be corrected as SL. It is clearly a typo since in all H1sequences, there is only one NL included in the conserved peptide GNLIAP located in the head of a spike.

Thirdly, we wish to address the question how to obtain the sequences corresponding to each Hk in tables S14 and S15. In fact, for each dataset corresponding to a fragment mentioned above, we select the subsets according to H1-H16 after all fragment sequences have been completely aligned using MCABMSA. We then compute the composition site by site. If on a site, all amino acids are the same or at least 99% of amino acids on this site are the same, we consider this site to be conserved (or invariant) and use the common amino acid as the element of the kind Hk at this given site. Otherwise, we let the largest two or three amino acids on this given site as the element of the Hk at the given site. Consequently, we find on some sites that there are two or thee amino acids separated by the symbol “/”.

After the comparison is made, we see that tables S14 and S15 are just an extension of table S13. Therefore, the binding site of CR6261 is a subset of all of invariant peptides (to different degrees) in these four fragments. Following from Tables S14 and S15, we clearly understand all of invariant peptides (to different degrees) in these four fragments.

1. **The table of the distribution of non-covalent bonds**

Table S16. The distribution of non-covalent bonds of the drugs on 3gbn, 3gbm, 3ztn, 3ztj, 3fku, 3sdy

| 3gbn | | ligand | a+b | a | b | Min{a,b} | | --- | --- | --- | --- | --- | | Azithromcyin | 36 | 23 | 13 | 13 | | Oseltamivir | 17 | 13 | 4 | 4 | | Zanamivir | 17 | 13 | 4 | 4 | | HEM | 27 | 9 | 18 | 9 | | Aspirin | 24 | 24 | 0 | 0 | | Isosorbide | 21 | 20 | 1 | 1 | | Vancomycin | 19 | 16 | 3 | 3 | | Amantadine | 9 | 8 | 1 | 1 | | Heroin | 18 | 18 | 0 | 0 | |
| --- | --- | --- | --- | --- | --- | --- | --- | --- | --- | --- | --- | --- | --- | --- | --- | --- | --- | --- | --- | --- | --- | --- | --- | --- | --- | --- | --- | --- | --- | --- | --- | --- | --- | --- | --- | --- | --- | --- | --- | --- | --- | --- | --- | --- | --- | --- | --- | --- | --- | --- | --- |
| 3gbm | | ligand | a+b | a | b | Min{a,b} | | --- | --- | --- | --- | --- | | Azithromcyin | 27 | 6 | 21 | 6 | | Oseltamivir | 25 | 0 | 25 | 0 | | Zanamivir | 20 | 0 | 20 | 0 | | HEM | 33 | 0 | 33 | 0 | | Aspirin | 14 | 0 | 14 | 0 | | Isosorbide | 14 | 0 | 14 | 0 | | Vancomycin | 26 | 2 | 24 | 2 | | Amantadine | 20 | 0 | 20 | 0 | | Heroin | 30 | 0 | 30 | 0 | |
| 3ztn | | ligand | a+b | a | b | Min{a,b} | | --- | --- | --- | --- | --- | | Azithromcyin | 24 | 17 | 7 | 7 | | Oseltamivir | 27 | 13 | 14 | 14 | | Zanamivir | 17 | 6 | 11 | 6 | | HEM | 26 | 7 | 19 | 7 | | Aspirin | 12 | 0 | 12 | 0 | | Isosorbide | 8 | 2 | 6 | 2 | | Vancomycin | 46 | 12 | 34 | 12 | | Amantadine | 15 | 15 | 0 | 0 | | Heroin | 25 | 4 | 21 | 5 | |
| 3ztj | | ligand | a+b | a | b | Min{a,b} | | --- | --- | --- | --- | --- | | Azithromcyin | 33 | 19 | 14 | 14 | | Oseltamivir | 28 | 14 | 14 | 14 | | Zanamivir | 15 | 10 | 5 | 5 | | HEM | 30 | 19 | 11 | 11 | | Aspirin | 17 | 12 | 5 | 5 | | Isosorbide | 17 | 15 | 2 | 0 | | Vancomycin | - | - | - | - | | Amantadine | 12 | 12 | 0 | 0 | | Heroin | 40 | 17 | 23 | 17 | |
| 3fku | | ligand | a+b | a | b | Min{a,b} | | --- | --- | --- | --- | --- | | Azithromcyin | 45 | 38 | 7 | 7 | | Oseltamivir | 20 | 20 | 0 | 0 | | Zanamivir | 23 | 23 | 0 | 0 | | HEM | 39 | 27 | 12 | 12 | | Aspirin | 15 | 15 | 0 | 0 | | Isosorbide | 18 | 18 | 0 | 0 | | Vancomycin | 30 | 24 | 6 | 6 | | Amantadine | 19 | 19 | 0 | 0 | | Heroin | 25 | 25 | 0 | 0 | |
| 3sdy | | ligand | a+b | a | b | Min{a,b} | | --- | --- | --- | --- | --- | | Azithromcyin | 3 | 0 | 3 | 0 | | Oseltamivir | 11 | 0 | 11 | 0 | | Zanamivir | 11 | 0 | 11 | 0 | | HEM | 7 | 0 | 7 | 0 | | Aspirin | 10 | 0 | 10 | 0 | | Isosorbide | 8 | 0 | 8 | 0 | | Vancomycin | 13 | 0 | 13 | 0 | | Amantadine | 7 | 0 | 7 | 0 | | Heroin | 7 | 0 | 7 | 0 | |

Table S17. The distribution of non-covalent bonds of the drugs on 3gbn, 3gbm, 3ztn, 3ztj, 3fku, 3sdy under some associated fashion

| 3gbn | | ligand | a1+b1 | a1 | b1 | Min{a1, b1} | | --- | --- | --- | --- | --- | | Azithromcyin | 36 | 23 | 13 | 13 | | **Oseltamivir** | **26** | **16** | **10** | **10** | | **Zanamivir** | **12** | **7** | **5** | **5** | | HEM | 37 | 11 | 26 | 11 | | Aspirin | 21 | 9 | 12 | 9 | | Isosorbide | 14 | 2 | 12 | 2 | | Vancomycin | 43 | 10 | 33 | 10 | | Amantadine | 16 | 7 | 9 | 7 | | Heroin | 18 | 9 | 9 | 9 | |
| --- | --- | --- | --- | --- | --- | --- | --- | --- | --- | --- | --- | --- | --- | --- | --- | --- | --- | --- | --- | --- | --- | --- | --- | --- | --- | --- | --- | --- | --- | --- | --- | --- | --- | --- | --- | --- | --- | --- | --- | --- | --- | --- | --- | --- | --- | --- | --- | --- | --- | --- | --- |
| 3gbm | | ligand | a1+b1 | a1 | b1 | Min{a1, b1} | | --- | --- | --- | --- | --- | | Azithromcyin | 24 | 4 | 20 | 4 | | **Oseltamivir** | **24** | **12** | **12** | **12** | | **Zanamivir** | **12** | **7** | **5** | **5** | | HEM | 15 | 6 | 9 | 6 | | Aspirin | 29 | 20 | 9 | 9 | | Isosorbide | 17 | 10 | 7 | 7 | | Vancomycin | 25 | 2 | 23 | 2 | | Amantadine | 44 | 29 | 15 | 15 | | Heroin | 12 | 7 | 5 | 5 | |
| 3ztn | | ligand | a1+b1 | a1 | b1 | Min{a1, b1} | | --- | --- | --- | --- | --- | | Azithromcyin | 21 | 11 | 10 | 10 | | **Oseltamivir** | **37** | **16** | **21** | **16** | | **Zanamivir** | **36** | **13** | **23** | **13** | | HEM | 27 | 18 | 9 | 9 | | Aspirin | 19 | 8 | 11 | 8 | | Isosorbide | 15 | 8 | 7 | 7 | | Vancomycin | 37 | 21 | 16 | 16 | | Amantadine | 21 | 14 | 7 | 7 | | Heroin | 37 | 19 | 18 | 18 | |
| 3ztj | | ligand | a1+b1 | a1 | b1 | Min{a1, b1} | | --- | --- | --- | --- | --- | | Azithromcyin | 37 | 22 | 15 | 15 | | **Oseltamivir** | **27** | **13** | **14** | **13** | | **Zanamivir** | **42** | **24** | **18** | **18** | | HEM | 27 | 16 | 11 | 11 | | Aspirin | 14 | 8 | 6 | 6 | | Isosorbide | 18 | 7 | 11 | 7 | | Vancomycin | 55 | 35 | 20 | 20 | | Amantadine | 16 | 7 | 9 | 6 | | Heroin | 33 | 23 | 10 | 10 | |
| 3fku | | ligand | a1+b1 | a1 | b1 | Min{a1, b1} | | --- | --- | --- | --- | --- | | Azithromcyin | 13 | 7 | 6 | 6 | | **Oseltamivir** | **31** | **20** | **11** | **11** | | **Zanamivir** | **13** | **6** | **7** | **6** | | HEM | 42 | 19 | 23 | 19 | | Aspirin | 30 | 21 | 9 | 9 | | Isosorbide | 20 | 13 | 7 | 7 | | Vancomycin | 25 | 18 | 7 | 7 | | Amantadine | 31 | 23 | 8 | 8 | | Heroin | 30 | 15 | 15 | 15 | |
| 3sdy | | ligand | a1+b1 | a1 | b1 | Min{a1, b1} | | --- | --- | --- | --- | --- | | Azithromcyin | 11 | 7 | 4 | 4 | | **Oseltamivir** | **21** | **16** | **5** | **5** | | **Zanamivir** | **14** | **7** | **7** | **7** | | HEM | 16 | 3 | 13 | 3 | | Aspirin | 20 | 15 | 5 | 5 | | Isosorbide | 11 | 5 | 6 | 5 | | Vancomycin | 15 | 10 | 5 | 5 | | Amantadine | 12 | 3 | 9 | 3 | | Heroin | 11 | 5 | 6 | 5 | |

1. **How to confirm the diversity for the pose of a drug docking with its benchmark pocket**

ADP is a famous liagand which may bind to many hundreds proteins and on some individual, it may also binds to many pockets. These results may be looked for from PDB database. For studying the inverse docking, we frequently require the similarity between proteins is less than 40% so that we can move the redundancy samples. Occasionally, we find that ADP may bind to many groups of proteins. In each group, the proteins are perfect same. For example, the first group of 12 proteins which may be bonded by ADP are 1x88_A, 1yrs_A, 2fky_B, 2fl2_A, 2fl6_A, 2g1q_B, 2q2y_A, 2q2z_B, 2wog_A, 2x7d_A, 2x7e_B and 3cjo_A. The second group of 11 proteins which may be bonded by ADP are 1bmf_D, 1e1q_D, 1e1r_D, 1efr_D, 2ck3_D, 2jiz_D, 2jj1_D, 2jj2_K, 2v7q_F, 2w6e_D and 2wss_M. For each of the protein, the pose of ADP binding to the protein was measured by x-ray method.

Therefore, for the 12 poses of ADP binding to the 12 proteins, we regard they are the 12 experimental results using the same proteins but independently repeat 12 times. We check whether or not these 12 poses are coincided. Typically, we use 1x88_A as the reference, and using the 3D alignment to compare each of the 12 proteins with 1x88_A, then we get the new coordinates of the 12 poses as the 2fky_B, 2fl2_A, 2fl6_A, 2g1q_B, 2q2y_A, 2q2z_B, 2wog_A, 2x7d_A, 2x7e_B and 3cjo_A are coincided to 1x88_A respectively. We may directly read the coordinates of the 11 “new poses” are different from the coordinates of the original pose of ADP binding to 1x88_A. For save space, we do not list all coordinates of the 12 poses. Alternatively, we load all 12 “now poses” and the original pose on 1x88_A together, and then we may find the union of total 12 poses is much bigger (figure S8 (A)). With same way, we use 1bmf_D as the reference, and align each of the 10 proteins 1e1q_D, 1e1r_D, 1efr_D, 2ck3_D, 2jiz_D, 2jj1_D, 2jj2_K, 2v7q_F, 2w6e_D and 2wss_M to get the coordinates of the 11 new poses, then we may also find these 11 poses of ADP binding to 1bmf_D are different. We also load all 11 poses on 1bmf_D, and then we also find the union of the 11 poses is much bigger (figure S8 (B)).


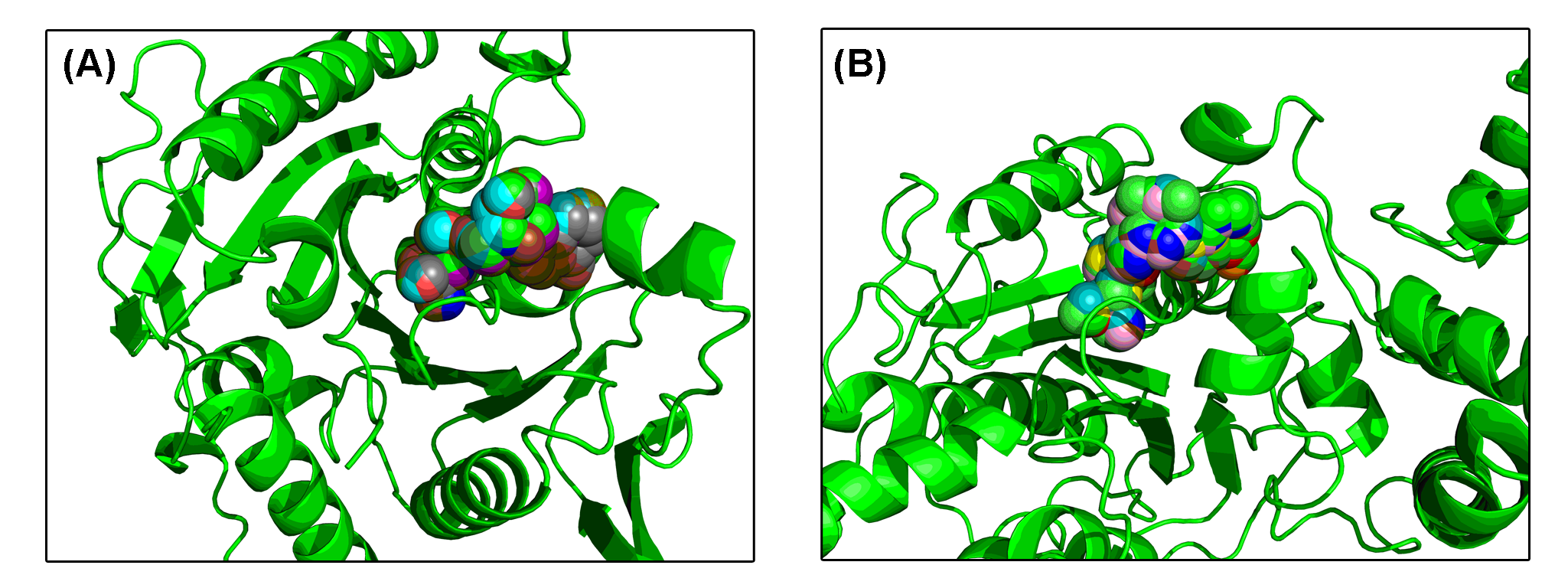


Figure S8. (A) The union of 12 poses binding to protein 1x88_A. (B) The union of 11 poses binding to protein 1bmf_D.

This comparison tells us that a ligand binding to its benchmark pocket does not obey the unique pose. The small error is permitted. That is, each pose in a neighbor of a real pose may happen in practice. Of curse, we may provide more examples because there are many ligands which may bind to lots of proteins may be found in PDB. For example, HEM, NAG and PLM also have many the multiple target proteins, we can use them to do the same thing.
